# G6PD deficiency in Indonesia: a systematic review and update of prevalence and variant maps in the context of malaria elimination

**DOI:** 10.1016/j.lanwpc.2026.101840

**Published:** 2026-03-25

**Authors:** Arkasha Sadhewa, Lydia Visita Panggalo, Illene Nanine, Ihsan Fadilah, Jontari Hutagalung, Enny Kenangalem, Prisca Cynthia Limardi, Safarina G. Malik, Rintis Noviyanti, Ayodhia Pitaloka Pasaribu, Jeanne Rini Poespoprodjo, Hellen Dewi Prameswari, Ric N. Price, Riskha Tiara Puspadewi, Wuryantari Setiadi, Leily Trianty, Herawati Sudoyo, Inge Sutanto, Din Syafruddin, J. Kevin Baird, Benedikt Ley, Iqbal R.F. Elyazar, Ari Winasti Satyagraha

**Affiliations:** a Global and Tropical Health Division, Menzies School of Health Research and Charles Darwin University, Darwin, NT, Australia; b Host Genetics Unit, EXEINS Health Initiative, Jakarta, Indonesia; c Oxford University Clinical Research Unit Indonesia, Faculty of Medicine, Universitas Indonesia, Jakarta, Indonesia; d Centre for Tropical Medicine and Global Health, Nuffield Department of Medicine, University of Oxford, Oxford, United Kingdom; e National Institute of Health Research and Development, Ministry of Health Republic of Indonesia, Jakarta, Indonesia; f Timika Malaria Research Program, Papuan Health and Community Development Foundation, Timika, Indonesia; g Genome Diversity and Disease Division, Mochtar Riady Institute for Nanotechnology, Tangerang, Indonesia; h Eijkman Research Center for Molecular Biology, National Research and Innovation Agency, Cibinong, Indonesia; i Department of Pediatrics, Medical Faculty, Universitas Sumatera Utara, Medan, Indonesia; j Mimika District Hospital, Timika, Indonesia; k Paediatric Research Office, Department of Child Health, Faculty of Medicine, Public Health and Nursing, Universitas Gadjah Mada, Yogyakarta, Indonesia; l Malaria Working Group, Ministry of Health Republic of Indonesia, Jakarta, Indonesia; m Mahidol-Oxford Tropical Medicine Research Unit, Faculty of Tropical Medicine, Mahidol University, Bangkok, Thailand; n Department of Parasitology, Faculty of Medicine, Universitas Indonesia, Jakarta, Indonesia; o Department of Parasitology, Faculty of Medicine, Universitas Hasanuddin, Makassar, Indonesia; p Hasanuddin University Medical Research Center (HUMRC), Universitas Hasanuddin, Makassar, Indonesia; q Division of Education, Menzies School of Health Research and Charles Darwin University, Darwin, NT, Australia

**Keywords:** G6PD deficiency, Plasmodium vivax, Malaria, Radical cure, Prevalence, Indonesia, Glucose-6-phosphate dehydrogenase, 8-Aminoquinoline, Primaquine, Tafenoquine

## Abstract

**Background:**

Low-daily-dose primaquine (PQ) (0·25 mg/kg/day over 14 days) remains the first-line treatment for *P. vivax* hypnozoites in Indonesia but can trigger haemolysis in glucose-6-phosphate dehydrogenase (G6PD) deficient individuals. Indonesia's malaria treatment guidelines do not require G6PD deficiency (G6PDd) screening prior to administering low-daily-dose PQ regimen, but future implementation of high-daily-dose PQ regimen (1 mg/kg/day over 7 days) will require G6PD screening. To date, no exhaustive assessment of G6PDd prevalence has been done in Indonesia.

**Methods:**

A systematic search of the literature was conducted (PROSPERO 2022 CRD42022368319). Studies meeting predefined criteria reporting G6PDd prevalence and genetic variants in Indonesia were identified in a systematic search and complemented with previously unpublished studies meeting the same criteria. The collected data are presented descriptively and geospatially mapped.

**Findings:**

A total of 45 studies published between 1964 and 2024 were included. The prevalences of G6PDd (<30% activity) were 0·0–19·9% across 87 sites (n = 23,166), and the prevalences of females with deficient and intermediate (30–70% activity) activities were 0·8–44·6% across 35 sites (n = 6729). G6PDd allele frequencies (males with <30% activity) were 0·0–25·9% across 82 sites (n = 10,680). Fifteen class B G6PD variants were reported, presenting oxidant-induced acute haemolytic anaemia. No relevant data were available for many areas of the country, including those with high *P. vivax* malaria incidences.

**Interpretation:**

Our findings support the introduction of routine G6PDd screening to guide high-daily-dose PQ treatment. However, G6PDd prevalence is heterogeneous across Indonesia, and available information is not comprehensive. This is of concern in areas with high endemicity of *P. vivax* malaria, where treatment with PQ is required. This lack of data needs to be addressed to inform and guide appropriate routine G6PDd screening to support *P. vivax* malaria elimination targets.

**Funding:**

This work was supported, in whole or in part, by the Bill & Melinda Gates Foundation (INV-024389).

**Research in context**

**Evidence before this study**

Primaquine (PQ) is currently the only hypnozoitocide used in radical cure treatment against *Plasmodium vivax* malaria relapse in Indonesia, but it may trigger haemolysis in patients with G6PD deficiency (G6PDd). Previously, G6PDd prevalence and genetic variants in Indonesia have been summarised and mapped as part of a global mapping project in 2012 and 2013, but no within-country summary has been generated, which is relevant to making informed decisions about the implementation of G6PD testing to support *P. vivax* malaria treatment. We confirmed this during the conception of the study by performing a systematic search on PubMed for studies published from database inception to October 5th, 2021, with the search term “((G6PD) OR (glucose 6 phosphate dehydrogenase)) AND (Indonesia)” and no language restrictions. The search returned 84 studies, published between 1963 and 2021 and all written in English, some of which reported G6PDd prevalence or variants, but none systematically summarised or reviewed the available evidence.

**Added value of this study**

This study systematically searched and screened published studies and theses from international and Indonesian repositories and combined them with additional data from 14 unpublished surveys. This study revealed the variation in G6PDd prevalence among Indonesian study sites and collated evidence of at least 15 different G6PD genetic variants with potential for PQ-induced haemolysis, and presented them in maps overlaying *P. vivax* incidence data. This study also highlighted gaps in geographical G6PDd data, including in areas of high *P. vivax* endemicity.

**Implications of all the available evidence**

Further collection of local evidence of G6PDd in high *P. vivax*-endemic areas is needed and will provide important considerations on the risks of implementing various PQ regimens to treat *P. vivax* malaria.

## Introduction

*P. vivax* is estimated to have caused 9·9 million malaria cases worldwide in 2024,^1^ the second largest malaria burden after *Plasmodium falciparum*. This burden is sustained by *P. vivax*'s ability to form dormant liver-stage parasites (hypnozoites) that can cause recurrent episodes of parasitaemia (relapses) weeks to months after infection.^2^ Difficulties in detecting and removing these hypnozoites make the elimination of *P. vivax* particularly challenging. In 2024, 257,070 cases of mono- or mixed *P. vivax* malaria were reported in Indonesia, accounting for half of the country's total reported cases of malaria.^1^ The Ministry of Health of the Republic of Indonesia is committed to eliminating endemic malaria by 2030,^3^ followed by the prevention of resurgence and to sustain a malaria-free status by 2045.^4^

Primaquine (PQ) and tafenoquine (TQ) are the only licensed drugs capable of eliminating hypnozoites.^2^ These drugs are used in combination with schizontocidal drugs for the radical cure of patients with *P. vivax* malaria. In Indonesia, the standard treatment for uncomplicated *P. vivax* malaria is a combination of blood schizontocidal drugs (dihydroartemisinin-piperaquine (DHP) over 3 days) plus a hypnozoitocidal agent (low-daily-dose PQ: 0·25 mg/kg body weight daily over 14 days).^5^ The treatment for patients with *P. vivax* malaria relapse is an intermediate-daily-dose PQ (0·5 mg/kg body weight daily over 14 days), and a weekly dose is given to those with known or suspected cases of glucose-6-phosphate dehydrogenase (G6PD) deficiency (0·75 mg/kg body weight weekly PQ given over 8 weeks).^5^

The active metabolites of PQ and TQ are strong oxidants known to induce lysis of red blood cells (haemolysis) in individuals with low G6PD enzyme activities, collectively called G6PD deficiency (G6PDd).^6^ In mature red blood cells (RBCs), G6PD is the sole electron donor for NADPH production, essential for cellular survival and the only means to compensate for oxidative stressors.^6^ G6PDd is caused by genetic variants of the *G6PD* gene (Xq28) that introduce functional instability, resulting in reduced enzymatic activities. G6PDd is a common X-linked enzymopathy^7^: hence males are either hemizygous normal or hemizygous deficient, whereas females can be homozygous normal, homozygous deficient, or heterozygous deficient. The reduction of enzymatic activity depends on the underlying genetic variants^6^ and in females with heterozygous G6PD alleles on random X-chromosome inactivation (lyonisation) as well.^7^ G6PDd is commonly diagnosed by measuring phenotypic enzyme activity from whole blood.^8^

The WHO recommends testing for G6PDd to guide the treatment of *P. vivax* malaria.^9^ Prescribing radical cure treatment without G6PD testing exposes individuals with G6PDd to the risk of severe haemolysis. Conversely, withholding the treatment due to concerns of drug-induced haemolysis puts patients with *P. vivax* malaria at risk of multiple relapses, and parasite-induced haemolysis associated with a cumulative risk of severe anaemia, attributable morbidity and mortality, and significant economic burden.^10^, ^11^, ^12^ In Indonesia, the greatest burden of malaria is in remote and poorly resourced areas where implementing G6PD testing prior to antimalarial treatment is challenging.^13^ Knowledge of a patient's G6PD status is critical to avoiding potentially life-threatening drug-induced haemolysis,^14^ especially in the aforementioned setting where access to urgent care may be limited.

Indonesia's malaria treatment guideline does not require G6PD testing prior to administering low-daily-dose PQ, but requires G6PD testing prior to the administration of intermediate-daily-dose PQ for patients with *P. vivax* malaria relapse (defined as recurrence of *P. vivax* malaria infection 28 days after radical cure treatment or later). There is no specific policy on the practice of G6PD testing and no definition of normal or deficient G6PD activity, even though the treatment dose for G6PD deficient individuals is clearly defined.^5^ Research into implementing high-daily-dose PQ regimen (1 mg/kg body weight daily over 7 days) has been prioritised by the Indonesian Ministry of Health to facilitate better efficacy and adherence,^3^ but this will require a more rigorous strategy for G6PD testing.^9^

Although G6PDd in Indonesia was first reported in the 1960s, there has been no comprehensive review of the available data on its prevalence and associated variants. This study collates available information on the prevalence and variant distribution of G6PDd across Indonesia.

## Methods

### Overview

A systematic review of the literature was conducted to identify published studies documenting the prevalence of G6PDd and known variants in Indonesia (PROSPERO 2022 CRD42022368319). In addition, unpublished data were identified through social networking. This systematic review of prevalence data followed the preferred reporting items for systematic reviews and meta-analyses (PRISMA) guidelines (Appendix pp. 2–3).

### Systematic review of the literature

PubMed was searched for relevant publications using the search terms “G6PD” and “Indonesia”. The Indonesia specific databases Rama, Garuda, OneSearch by Perpusnas, and Neliti were searched for studies with the key words “G6PD” or “Glucose-6-phosphate dehydrogenase” or “Glukosa-6-fosfat dehidrogenase”. Publications used by the Malaria Atlas Project (MAP) as references for Indonesian G6PDd prevalence data were added to the search results.^15^ The search was last performed on June 28th, 2024, and the titles and abstracts of identified studies were screened for eligible publications: those reporting G6PD activity of human participants in Indonesia from any year and written in English or Bahasa Indonesia were considered. Following the screening of their titles and abstracts, relevant publications were further screened by full text. Studies reporting the prevalence of G6PDd using qualitative, quantitative, or molecular assays in populations representative of the study sites were considered; participant representativeness here defined as residing permanently (5 years, or since birth for participants aged ≤5 years) at the respective study site. For studies with qualitative and quantitative G6PD assays, case–control, case report, and meta-analysis studies were excluded, as well as studies with purposive sampling based on ethnicity or blood disorder, and studies with participants aged below 3 months. Studies with quantitative G6PD assays and fewer than 35 male participants were also excluded. For studies using molecular assays to identify G6PDd, only meta-analysis study design was excluded from consideration. Screening was done by two authors blinded to the results of the other author. In case of contradictory findings, an additional screening was undertaken by a third author, and this was considered final. Criteria for the systematic screening (Appendix p. 4) were also applied to datasets from studies performed by the Eijkman Institute for Molecular Biology's Red Blood Cell Membrane and Enzyme Disorders Unit and its collaborators. All included studies reporting prevalence data were assessed for quality using the Joanna Briggs Institute Critical Appraisal Checklist (Appendix pp. 5–7).^16^ A funnel plot was generated and tested for asymmetry with the Egger's test to investigate publication bias among included studies.

### Data collection

The corresponding authors of eligible publications using quantitative G6PD measurements were contacted and requested to provide individual participant data (IPD). Essential data were extracted from available IPD, including study location, G6PD activity, G6PD activity measurement method, haemoglobin (Hb) level, Hb measurement method, and sex of participant. Desirable data included G6PD variant data and the genotyping method. When only aggregate data were available, aggregated G6PDd prevalence, G6PD variant, study location, assays employed, and participants’ sex data reported in the publication were extracted.

### Outcomes

The primary outcomes of this study were descriptive maps of G6PDd prevalence, G6PDd allele frequency, and combined prevalence of G6PD deficient and intermediate females, and of G6PD variants in Indonesia, superimposed over *P. vivax* incidence map. Additional outcomes were a model-based map predicting the prevalence of G6PDd across Indonesia, and estimations of the country's overall G6PDd prevalence, G6PDd allele frequency, and the combined prevalence of G6PD deficient and intermediate females.

### Data processing

In studies using qualitative G6PD testing and in studies with quantitative testing where IPD were not available, study-specific definitions of G6PDd reported in the corresponding article were used. In these cases, aggregated data on G6PDd prevalence were used in the main analysis.

In studies where quantitative G6PD testing was reported and IPD were available, the adjusted male median (AMM) was calculated for each study and defined as 100% of G6PD activity.^17^ For multi-centre studies, the AMM was calculated for each site. If results from multiple studies were available for the same site, the AMM was calculated for each study separately, and the respective study population was categorised on the study-specific AMM. The prevalence of G6PDd was then pooled across all studies from the respective sites.

### Statistical analysis

G6PDd was defined as an activity of less than 30% of the study-or-site-specific AMM. Females with activities between ≥30% and <70% were categorised as having intermediate activity,^9^ and all others were classified as G6PD normal. To calculate G6PDd prevalence, only individuals with less than 30% activity were considered; all others were considered G6PD normal. Allele frequencies, which in X-linked disorders are equivalent to prevalence in males, were calculated from G6PD deficient males only. In a sub-analysis, only female participants from studies where quantitative G6PD testing was reported and IPD were available were considered, and any G6PD activity <70% of the AMM was defined as G6PD deficient. Individuals with anaemia (Hb <8·0 g/dL) were excluded from AMM calculations and all subsequent analyses.

The overall G6PDd prevalence, G6PDd allele frequency, and the combined prevalence of G6PD deficient and intermediate females were estimated by performing a two-stage IPD meta-analysis for a single proportion using the meta suite from STATA. The effect sizes, defined as the Freeman-Tukey transformed proportions, were calculated for each study with available and applicable IPD, and were combined with a random-effects model with the REML (restricted maximum likelihood) estimation method. Sensitivity analysis was performed for each estimate by doing a leave-one-out meta-analysis. For the estimated G6PDd prevalence, a subgroup analysis was performed to investigate heterogeneity considering the proportion of male participants at each site (0–25%, 26–50%, 51–75%, or 76–100%), the decade the study was published (or conducted for unpublished studies; 1964–1969, 1980–1989, 1990–1999, 2000–2009, 2010–2019, or 2020–2024), the assay (quantitative spectrophotometry, qualitative WST, qualitative MTT/PMS, or other qualitative methods; Appendix pp. 9–17), and the province (administrative level 1) where the study site is located. All the above data processing and analysis were done using STATA version 18 (StataCorp, College Station, TX, USA).

### Mapping

*P. vivax* annual parasite incidence (PvAPI) data per city/regency was calculated from the 2023 *P. vivax* annual case count provided by the Indonesian Ministry of Health and the projected 2023 city/regency (administrative level 2) population based on the 2020 Indonesia Population Census.^18^ PvAPI data per city/regency were mapped into Indonesia city/regency border shapefile^19^ using R (version 4.4.1) and RStudio (version 2024.09.0.375) with R packages: sf, ggplot2, dplyr, and ggnewscale. Study- or site-specific G6PDd prevalence data, allele frequency data, and data of females with G6PD activity <70% were superimposed on the PvAPI map using the same packages. Studies with no available individual or aggregated participant sex data were excluded from maps requiring sex-disaggregated data.

Pie charts of G6PD variant data from population-based studies were placed over the PvAPI map with the aforementioned R packages and the scatterpie R package. Only variant data from individuals classified as G6PD deficient were mapped, and variant data from studies performed in the same city/regency were grouped together into the same pie chart. G6PD variant data from non-population-based studies (case reports, family studies, studies with only G6PD deficient participants) were reported in a tabular format.

A geostatistical model-based predictive map of mean G6PDd prevalence was created using the R-INLA package: the observed study-specific G6PDd prevalence values were specified as response vectors at the corresponding sites' locations, and the model y ∼ 0 + b0 + f (s, model = spde) was used to fit mean predicted G6PDd prevalence values over a mesh covering the surface of the map of Indonesia (y), with the intercept (b0) as the sole fixed effect and f (s, model = spde) represents the spatial random effect (s), modelled as the gaussian random field using Stochastic Partial Differential Equations (SPDE) approach. The model was fitted using the inla( ) function, with a binomial likelihood family linked with the logit function and using the default priors. The 2·5th and 97·5th percentiles (upper and lower limits of 95% credible intervals) of the fitted values for all prediction locations were mapped to illustrate the uncertainty of the predictive values. The prior mean and prior precision of the hyperparameters (θ) of the SPDE model were manually reconfigured post-analysis to adjust the scale and variance of the modelled Gaussian random field. The model's fit was assessed by computing the Conditional Predictive Ordinance (CPO) and Probability Integral Transform (PIT) values for each observation.

### Role of the funding source

The study funders had no role in study design, data collection, data analysis, interpretation, or writing of the manuscript.

## Results

**Fig. 1:**
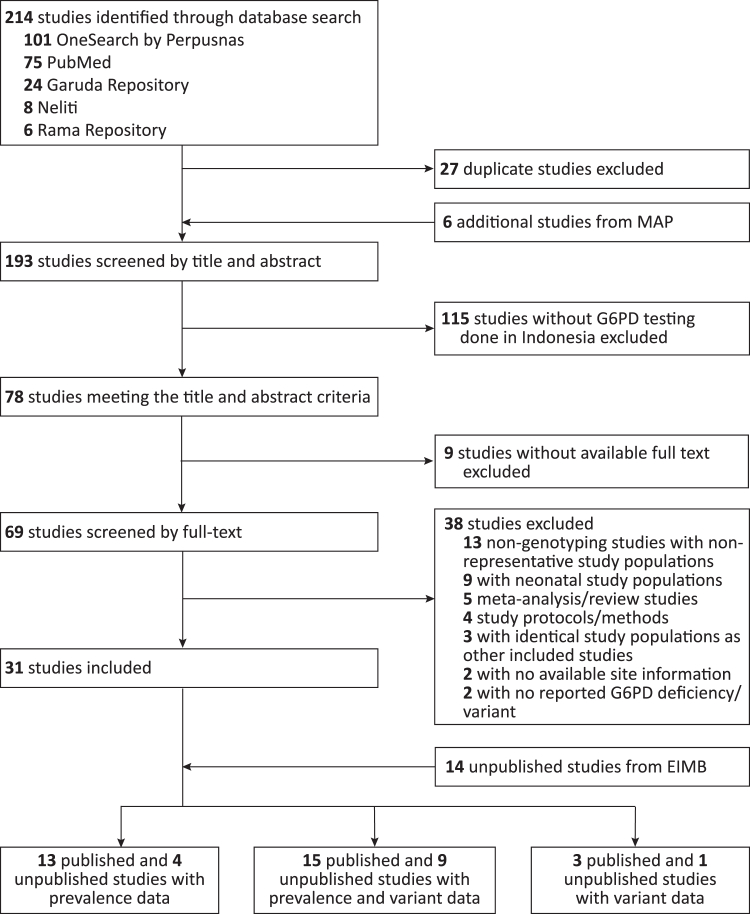
Diagram of the systematic search performed to gather representative studies reporting G6PDd prevalence data and all studies reporting G6PD variant data in Indonesia. MAP = the Malaria Atlas Project; EIMB = Eijkman Institute for Molecular Biology.

The systematic search identified 193 published studies, including six studies from the MAP's reference list. Based on the title and abstract, 115 (59·6%) studies not meeting the inclusion criteria were excluded (Appendix p. 4). Of the remaining 78 studies, nine (11·5%) were excluded because the corresponding full texts were not available. The full texts of 69 studies were screened, and 13 (18·8%) studies reporting G6PDd prevalence,^20^, ^21^, ^22^, ^23^, ^24^, ^25^, ^26^, ^27^, ^28^, ^29^, ^30^, ^31^, ^32^ 15 (21·7%) studies reporting prevalence and variants,^33^, ^34^, ^35^, ^36^, ^37^, ^38^, ^39^, ^40^, ^41^, ^42^, ^43^, ^44^, ^45^, ^46^, ^47^ and three (4·4%) studies reporting variants^14^^,^^48^^,^^49^ were included (Table 1 and Fig. 1). All studies included in the analysis were published between 1964 and 2024.

**Table 1:** Details of published studies reporting G6PDd prevalence and variant data from Indonesia included through the systematic search.

Article, year of publication	Province	City/regency	Site(s)	Number of site(s)	Assay type	n Participants (Male/Female)	Study population
Reporting prevalence data included in the prevalence, allele frequency, and predictive maps							
Eng, 1964^20^	DKI Jakarta	Jakarta Pusat	Jakarta	1	Qualitative	446 (446/0)	Healthy participants and hospital patients
Breguet, 1982^21^	Bali	Karangasem	Tenganan Pageringsingan Village	1	Qualitative	316 (166/150)	Residents aged >12 years
Matsuoka, 1986^22^	North Sumatra	Nias Utara, Nias Selatan, Batubara, Medan	Afia, Boto Hilitano, Hiliana'a, Durian, Perupuk & Guntung, and Medan	6	Qualitative	1147 (568/579)	Elementary school students
Jones, 1990^a^^,^^23^	Papua	Keerom	Arso PIR	1	Quantitative	223 (179/44)	Residents, including transmigrants from Java and native Papuan
Fryauff, 1995^24^	Papua	Keerom	Arso XI	1	Qualitative	131 (131/0)	Transmigrant residents from Java aged >15 years
Azhar, 1998^25^	East Nusa Tenggara, West Nusa Tenggara	Alor, Sumba Timur, Sumbawa	Alor, Sumba Timur, Sumbawa	3	Qualitative	348 (156/192)	High school students (majority) and healthy adults
Tantular, 1999^26^	North Maluku	Tidore Kepulauan	Oba, Oba Selatan, Siokona	3	Qualitative	1126 (554/572)	Survey volunteers
Azhar, 2001^27^	Aceh	Banda Aceh, Central Aceh	Syiah Kuala, Takengon	2	Qualitative	139 (88/51)	Healthy university and high school students (majority)
Syahyuni, 2003^28^	East Nusa Tenggara	Sumba Timur	Waingapu, Kambaniru, and Umalulu	1	Qualitative	210 (93/117)	Grade IV and V elementary school students
Jalloh, 2004^29^	East Java, East Nusa Tenggara	Surabaya, Sikka	Surabaya, Sikka	2	Qualitative	1286 (648/638)	Survey volunteers (Surabaya and Sikka); elementary school students and teachers (Sikka)
Shimizu, 2005^30^	East Nusa Tenggara	Sumba Timur	Sumba Timur	1	Qualitative	210 (100/110)	Healthy participants
Lederman, 2006^d^^,^^31^	Central Java	Purworejo	Menoreh Hills	1	Qualitative	124	Participants with uncomplicated *P. falciparum* malaria
Tuda, 2007^32^	North Sulawesi	Bitung, Bolaang-Mongondow, Minahasa Utara	Ranowulu, Lolak, Wori	3	Qualitative	442 (243/199)	Elementary school students
Reporting prevalence and variant data included in the prevalence, allele frequency, predictive, and variant maps							
Soemantri, 1995^33^	Central Java	Semarang	Semarang	1	Qualitative	169 (169/0)	Adult male participants
Davy, 2000^34^	Bangka Belitung Islands, Central Kalimantan, North Sumatra	Bangka, Palangkaraya, Medan	Bangka, Palangkaraya, Medan	3	Qualitative	117 (117/0)	Healthy male participants
Hardjowasito, 2001^d^^,^^35^	East Nusa Tenggara	Timor Tengah Utara	Insana	1	Qualitative	118	Randomly selected participants
Iwai, 2001^36^	Maluku and North Maluku	Buru and Halmahera	Buru and Halmahera	1	Qualitative	696 (696/0)	Population-based survey volunteers
Matsuoka, 2003^37^	East Nusa Tenggara	Sikka	Maumere and Talibura	1	Qualitative	363 (177/186)	Elementary school students
Kawamoto, 2006^38^	East Nusa Tenggara	Ende, Sikka	Ende, Maumere	2	Qualitative	1108 (642/466)	Febrile volunteers
Suhartati, 2006^39^	Maluku	Kepulauan Tanimbar, Maluku Barat Daya, Tual	Larat, Saumlaki, Pulau Babar, Pulau Romang, Pulau Kur	5	Qualitative	298 (144/154)	Visitors of a free community-service health clinic
Tantular, 2010^40^	East Nusa Tenggara, North Sulawesi, Southeast Sulawesi	Flores Timur, Manggarai Barat, Nagekeo, Ngada, Sikka, Sumba Timur, Timor Tengah Selatan, Minahasa, Minahasa Utara, Konawe, Muna	Larantuka, Labuan Bajo, Tonggo Village, Reo Village, Tiworiwu Village, Pruda Village, Reruwairere Village, Waingapu, Soe and Oebobo, Minahasa, Bangka Island, Lambuya Village, Muna Island	13	Qualitative	2777 (1657/1120)	Population-based survey volunteers
Asih, 2012^d^^,^^41^	Aceh	Sabang	Sabang	1	Qualitative	937	Population-based survey volunteers
Hutagalung, 2015 b^,^^c^^,^^42^	East Nusa Tenggara	Timor Tengah Selatan	Batu Putih, Oe'ekam, Oenino, Oinlasi, Panite	5	Quantitative	552 (227/325)	Population-based survey volunteers
Satyagraha, 2015^b^^,^^43^	East Nusa Tenggara	Sumba Barat, Sumba Barat Daya, Sumba Tengah	Lamboya, Wanokaka, Mali Mada, Mata Pyawu, Kodi, Umbu Ratu Nggay, Wairasa, Anakalang	8	Quantitative	1996 (833/1163)	Population-based survey volunteers (residents)
Satyagraha, 2016^b^^,^^44^	East Nusa Tenggara	Sumba Barat Daya	Panenggo Ede	1	Quantitative	607 (259/348)	Population-based survey volunteers (residents)
Satyagraha, 2021^b^^,^^45^	East Nusa Tenggara	Sumba Barat Daya	Kodi Balaghar, Umbu Ngedo	1	Quantitative	2028 (0/2028)	Female healthy volunteers
Sadhewa, 2024a^b^ and 2024b^b^^,^^46^^,^^47^	North Kalimantan	Malinau, Nunukan	Malinau, Nunukan	2	Quantitative	145 (45/100)	Individuals aged ≥6 years visiting community health centres
Reporting variant data included in the variant table							
Sulistyaningrum, 2020^48^	East Nusa Tenggara	Timor Tengah Selatan	Batu Putih, Oe'ekam, Oenino, Oinlasi, Panite	5	PCR-RFLP	381 (156/225)	Study participants with asymptomatic malaria, or G6PD activity <6·97 U/g Hb, or anaemia
Taylor, 2023^49^	Lampung, North Sumatra	Pesawaran, Labuhanbatu Utara	Hanura, Tanjung Leidong	2	PCR-RFLP, exon sequencing	19 (14/5)	Study participants with confirmed G6PDd
Kosasih, 2023^14^	North Sumatra, East Nusa Tenggara	Batubara, Sumba Barat Daya	Tanjung Tiram	2	PCR-RFLP, exon-sequencing	2 (2/0)	Acute haemolytic anaemia patients from malaria trials

PCR-RFLP = genotyping by polymerase chain reaction—restriction fragment length polymorphism.

a Studies with quantitative G6PD activity measurement, in which IPD was not obtained.

b Studies with quantitative G6PD activity measurement, in which IPD was obtained.

c Variant data included in the variant table instead of the variant map.

d Not Included in G6PDd Allele Frequency and G6PDd Prevalence in Females Maps due to lack of reported participant sex data.

In addition to the systematic search results, unpublished data from 14 studies conducted between 2011 and 2023 were also included (Appendix p. 8), of which four were studies of G6PDd prevalence, nine studies of G6PDd prevalence and variants, and one study with variant data only (Table 2).

**Table 2:** Details of previously unpublished studies reporting G6PDd prevalence and variant data from Indonesia meeting the systematic search's criteria.

Local Principal investigator(s), year of study	Province	City/regency	Site(s)	Number of site(s)	Assay type	n Participants (Male/Female)	Study population
Reporting prevalence data included in the prevalence, allele frequency, and predictive maps							
Sudoyo, 2015	Jambi	Batang Hari	Bukit Dua Belas National Park	1	Quantitative	239 (111/128)	Population-based survey, 1 individual per household
Sudoyo, 2016	West Sumatra	Kepulauan Mentawai	Mentawai	1	Quantitative	94 (94/0)	Population-based survey, 1 individual per household
Sudoyo, 2017	Maluku	Maluku Tengah	Seram Utara	1	Quantitative	55 (55/0)	Population-based survey, 1 individual per household
Poespoprodjo, 2018	Papua	Mimika	Timika	1	Quantitative	356 (181/175)	Population-based survey volunteers (residents)
Reporting prevalence and variant data included in the prevalence, allele frequency, predictive, and variant maps							
Satyagraha, 2012	South Kalimantan	Banjarmasin, Banjarbaru	Banjarmasin & Banjarbaru (combined)	1	Quantitative	201 (77/124)	Healthy vocational school students (residents)
Satyagraha, 2012	North Maluku	Halmahera Timur	Maba	1	Quantitative	140 (65/75)	Healthy middle school students (residents)
Satyagraha, 2013	Bangka Belitung Islands	Bangka, Bangka Tengah	Bangka, Central Bangka	2	Quantitative	606 (225/381)	Healthy population-survey volunteers (residents) aged >6 years old
Syafruddin & Setiadi, 2014	Central Kalimantan	Gunung Mas, Kapuas, Kotawaringin Timur, Barito Utara, Murung Raya	Gunung Mas, Pujon, Sei Pinang, Waringin Agung, Barito Utara, Murung Raya	6	Quantitative	1530 (845/685)	Population-based survey volunteers (residents)
Sutanto, Pasaribu, & Satyagraha, 2016	Lampung, North Sumatra	Pesawaran, Labuhanbatu Utara	Hanura, Tanjung Leidong	2	Quantitative	608 (183/425)	Individuals with fever/history of fever, visiting community health centres
Syafruddin, 2017	Bengkulu	Bengkulu Utara	Arga Makmur, Enggano	2	Quantitative	483 (149/334)	Population-based survey volunteers (residents)
Syafruddin, 2017	Papua	Keerom	Keerom	1	Quantitative	206 (56/160)	Population-survey (residents)
Noviyanti, 2018	East Nusa Tenggara	Timor Tengah Selatan	Boking	1	Quantitative	294 (165/129)	Population-based survey, 1 individual per household
Satyagraha, 2020	Papua	Mimika	Timika	1	Quantitative	295 (136/159)	Individuals with fever/history of fever, visiting Timika Jaya community health centre
Reporting variant data included in the variant table							
Malik, 2024^a^	East Nusa Tenggara, Maluku, South Papua	Manggarai, Sumba Timur, Kepulauan Tanimbar, Mappi	Cibal, Wunga, Sangliat Dol, Korowai	4	Whole-genome sequencing	98 (78/20)	Population survey, 1 individual per household (resident)

IPD was obtained for all the studies listed in this table.

a Samples collected between 2005 and 2016.

Site-specific G6PDd prevalence was calculated for 87 sites based on the G6PD status of 23,166 participants (Appendix pp. 9–17). At least one site was included in 22 out of the 38 provinces of Indonesia. At 38 out of 87 sites (43·7%), the AMM was determined from IPD G6PD measurements and ranged between 6·60–12·51 U/g Hb; with all measurements done by spectrophotometry using commercial kits from either Randox Laboratories (UK), Trinity Biotech (Ireland), or Pointe Scientific (USA) (Appendix pp. 9–17).

**Fig. 2:**
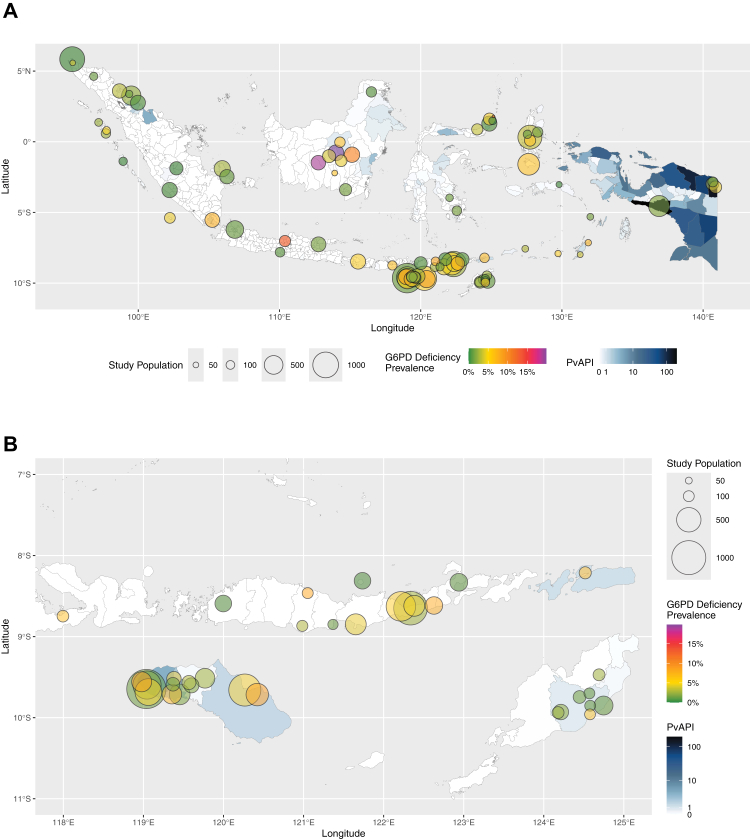
Site-specific G6PDd prevalence (green to purple shaded circles) superimposed on city/regency-level PvAPI choropleth map (blue shades) of Indonesia (A) and East Nusa Tenggara province (B). The size of each site's study population was indicated by circles on a continuous scale. G6PD = glucose-6-phosphate dehydrogenase; PvAPI = *Plasmodium vivax* annual parasite index.

The overall prevalence of G6PDd was 3·1% (95% confidence interval [CI]: 2·4–3·8%), with significant heterogeneity between the prevalence of individual sites (95% prediction interval [PI]: 0·0–10·8%; Appendix p. 21). The prevalence of G6PDd ranged from 0·0% to 19·9% (Appendix pp. 9–17 and Fig. 2). The highest prevalence of 19·9% (95% CI: 15·8–24·5%) was reported in Sei Pinang, Central Kalimantan. Four sites reported zero deficient cases: Bitung City (North Sulawesi; 95% CI: 0·0–4·6%), Bukit Dua Belas National Park (Jambi; 95% CI: 0·0–1·5%), Mentawai Island (West Sumatra; 95% CI: 0·0–3·9%), and Seram Island (Maluku; 95% CI: 0·0–6·5%). Significant heterogeneity remained within the subgroups analysed by proportion of male participants, decade of study, assay, and in the majority of prevalence estimates pooled at the province (administrative level 1) level (Appendix p. 22). The 2023 PvAPI in Indonesian cities/regencies ranged from 0·0 to 196·6 cases per 1000 population (Fig. 2).

**Fig. 3:**
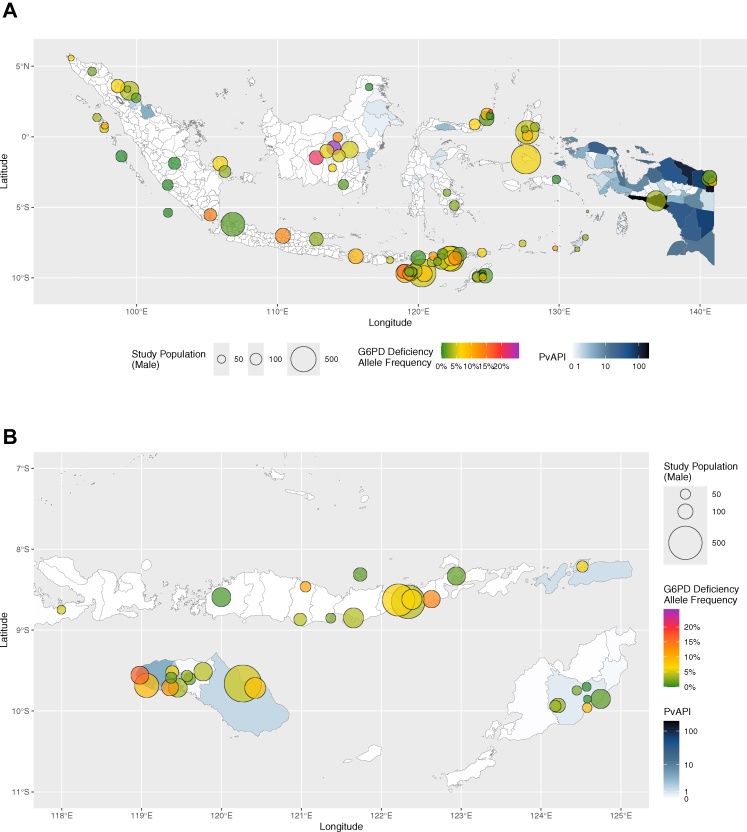
Site-specific G6PDd allele frequency (green to purple shaded circles) superimposed on city/regency-level PvAPI choropleth map (blue shades) of Indonesia (A) and East Nusa Tenggara province (B). The size of each site's study population was indicated by circles on a continuous scale. G6PD = glucose-6-phosphate dehydrogenase; PvAPI = *Plasmodium vivax* annual parasite index.

G6PDd allele frequencies were calculated from 10,680 male participants recruited at 82 sites. The overall allele frequency was 4·5% (95% CI: 3·5–5·5%) with frequencies from individual sites showing significant heterogeneity (95% PI: 0·0–15·0%; Appendix p. 23). Frequencies ranged from 0·0–25·9% (Appendix pp. 9–17 and Fig. 3). The highest allele frequency (25·9%; 95% CI: 19·0–33·7%) was observed in Sei Pinang, Central Kalimantan, and the lowest frequency of 0·0% was found in Bitung City (North Sulawesi; 95% CI: 0·0–7·1%), Oinlasi and Oe'ekam (East Nusa Tenggara; 95% CI: 0·0–10·0% and 0·0–8·4%, respectively), Malinau (North Kalimantan; 95% CI: 0·0–7·9%), Bukit Dua Belas National Park (Jambi; 95% CI: 0·0–3·3%), Mentawai Island (West Sumatra; 95% CI: 0·0–3·9%), Arga Makmur and Enggano Island (Bengkulu; 95% CI: 0·0–4·2% and 0·0–5·7%, respectively), and Seram Utara (Maluku; 95% CI: 0·0–6·5%).

**Fig. 4:**
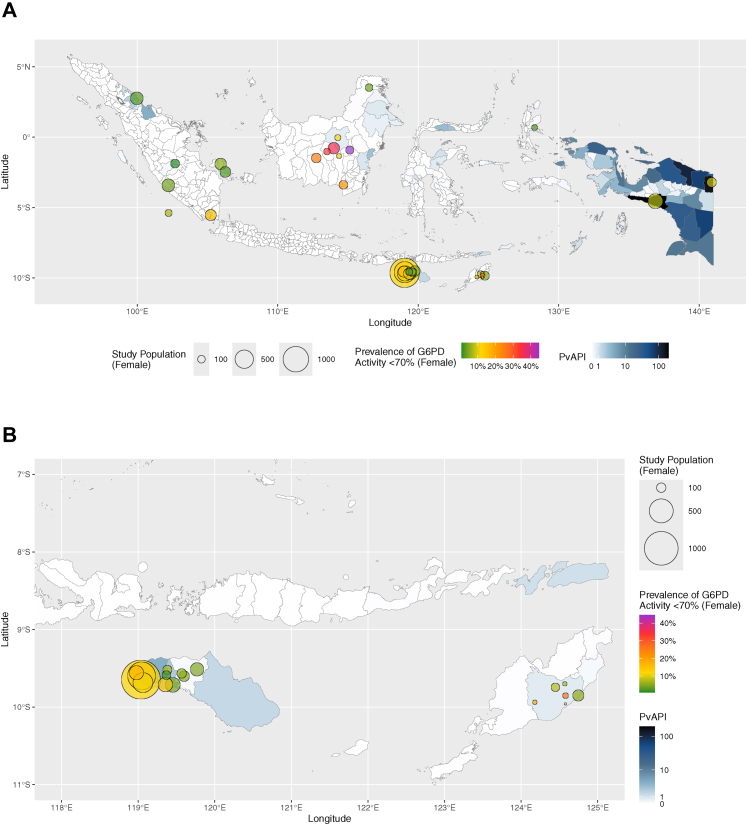
Site-specific prevalence of female participants with G6PD activity <70% of normal (green to purple shaded circles) superimposed on city/regency-level PvAPI choropleth map (blue shades) of Indonesia (A) and East Nusa Tenggara province (B). The size of each site's study population was indicated by circles on a continuous scale. G6PD = glucose-6-phosphate dehydrogenase; PvAPI = *Plasmodium vivax* annual parasite index.

The prevalence of female participants with G6PD activity <70% was calculated from 6729 female participants from 35 sites. The overall prevalence was 10·4% (95% CI: 7·6–13·7%), with significant heterogeneity between sites (95% PI: 0·0–33·9%; Appendix p. 24). The prevalences ranged from 0·8–44·6% (Appendix pp. 9–17 and Fig. 4). The highest prevalence of 44·6% (95% CI: 35·3–54·3%) was reported in Barito Utara, Central Kalimantan, and the lowest prevalence of 0·8% (95% CI: 0·2–4·3%) was found in Bukit Dua Belas National Park, Jambi.

There was no significant publication bias among the included studies (Egger's test p = 0·870; Appendix p. 25). Leave-one-out sensitivity analysis performed for each overall prevalence or allele frequency estimate confirmed that no single study exerted a significantly larger influence on the pooled estimates (Appendix pp. 26–28).

**Fig. 5:**
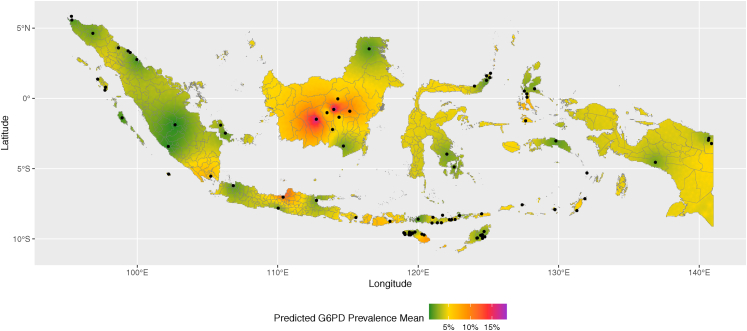
Geostatistical map of mean predicted G6PDd prevalence (green to purple area) in Indonesia modelled from site-specific G6PDd prevalence data. Black dots mark study sites. G6PD = glucose-6-phosphate dehydrogenase.

A continuous map of mean predicted G6PDd prevalence was generated from the site-specific G6PDd prevalence data using a geostatistical model (Fig. 5). The predicted G6PDd prevalence in areas immediately surrounding the study sites reflects the corresponding sites' reported prevalence. The uncertainty of the predicted prevalence was mapped as the lower and upper limits of 95% credible intervals (2·5th and 97·5th percentiles of the fitted values; Appendix p. 29). The assessment of the model's fit showed small CPO values and a non-uniform distribution of PIT values (Appendix p. 30).

**Fig. 6:**
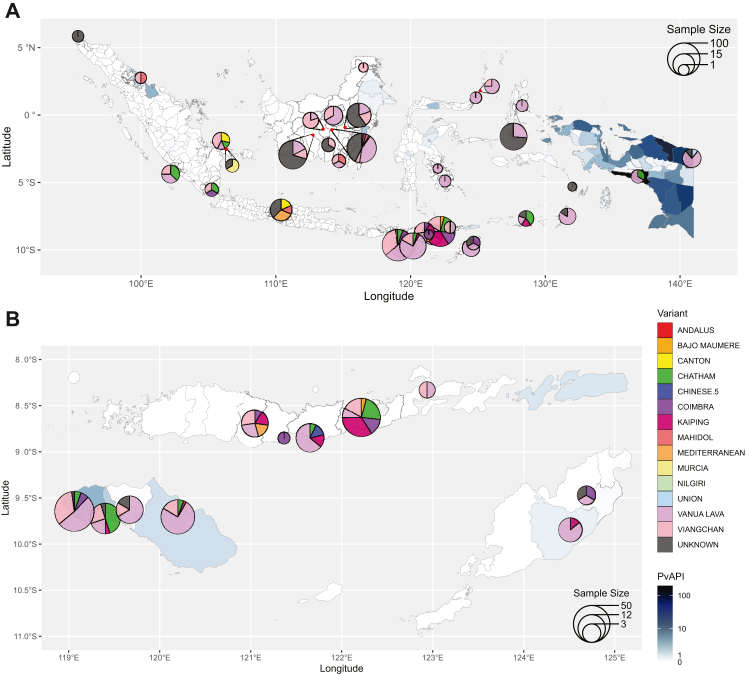
Study-and-regency-specific G6PD variant distribution superimposed on city/regency-level PvAPI choropleth map (blue shades) of Indonesia (A) and East Nusa Tenggara province (B), among individuals classified as G6PD deficient. Pie charts were positioned above the respective study sites, except where red dots are used to mark the study sites. Each site's sample size was indicated by circles on a continuous scale. PvAPI = *Plasmodium vivax* annual parasite index.

In total, samples from 542 G6PD deficient participants enrolled at 37 sites underwent molecular analysis to determine local G6PD variants. Fourteen clinically relevant G6PD variants were identified in 413 individuals (76·2%; Fig. 6 and Appendix pp. 18–20), all of which were categorised as Class B.^50^ The most frequently reported variant was Vanua Lava, which was reported from 199 individuals (48·2%) diagnosed with a known variant. Among all individuals genotyped, no G6PD variant was identified in 129 individuals (23·8%); 11 of these were genotyped by variant-specific PCR-RFLP, and 118 by various exon sequencing methods (Appendix pp. 18–20). Five non-population-based studies reported 39 individuals with known G6PD genetic variants (Table 3), all of whom had the same variants as the individuals included in the variant map, except one individual with the Orissa variant (Class B).

**Table 3:** G6PD variants reported in non-population-based studies (case series, studies with only G6PD deficient participants, and studies where genotyped participants were chosen by criteria other than G6PDd).

Site(s)	Study	Province	City/regency	G6PD variant (n)	Genotyping method	Study type
Oinlasi, Oe'ekam, Panite, Batu Putih, Oenino	Hutagalung, 2015^42^	East Nusa Tenggara	Timor Tengah Selatan	Vanua Lava (6)	PCR-RFLP	Population screening
	Sulistyaningrum, 2020^48^			Kaiping (2), Coimbra (16)	PCR-RFLP	Genotyping G6PD deficient and malaria-positive participants from Hutagalung, 2015 study
Hanura	Taylor, 2023^49^	Lampung	Pesawaran	Viangchan (3), Orissa (1), Chatham (1), Vanua Lava (1)	PCR-RFLP, sequencing	G6PD deficient only
Tanjung Tiram	Kosasih, 2023^14^	North Sumatra	Batubara	Coimbra (1)	PCR-RFLP	Case series
Sumba Barat Daya	Kosasih, 2023^14^	East Nusa Tenggara	Sumba Barat Daya	Vanua Lava (1)	NIR	Case series
Cibal	Malik, 2024	East Nusa Tenggara	Manggarai	Mediterranean (1)	Whole-genome sequencing	Population genetics study^a^
Wunga	Malik, 2024	East Nusa Tenggara	Sumba Timur	Chatham (1), Vanua Lava (3)	Whole-genome sequencing	Population genetics study^a^
Sangliat Dol	Malik, 2024	Maluku	Kepulauan Tanimbar	Vanua Lava (1)	Whole-genome sequencing	Population genetics study^a^
Korowai	Malik, 2024	South Papua	Mappi	Chatham (2)	Whole-genome sequencing	Population genetics study^a^

NIR = no information retrieved.

PCR-RFLP = genotyping by polymerase chain reaction—restriction fragment length polymorphism.

a Population study without G6PD phenotypic testing.

## Discussion

The prevalence of G6PDd in Indonesia is highly heterogeneous, ranging from zero to 20%, with 6% of the sites having a prevalence of greater than 10%, but the majority (64/87) of the surveyed sites reported a prevalence of less than 5% (Appendix pp. 9–17). There were significant gaps in available data, including highly endemic areas for *P. vivax* endemicity such as in Papua (Fig. 2). Considering the observed heterogeneity, gaps in available data, and the genetic diversity of the Indonesian population,^51^ the national-level estimates presented in this study should be interpreted with caution. Past efforts in mapping G6PDd presented national-level estimations of prevalence or allele frequency,^52^, ^53^, ^54^ none of which captured Indonesia's range of G6PDd prevalence reported in this study. The most recent predictive geospatial map of G6PDd^54^ was done on a global scale and thus lacked granularity when presenting estimated country-specific data. The Indonesian prevalence data were extracted from only 13 studies, and the national-level allele frequency was estimated at 7% (IQR: 5–9%) in Indonesia, which was higher than the estimated overall allele frequency in this study.

Significant heterogeneity was also observed when investigating study-level covariates (Appendix p. 22), reflecting the inherent heterogeneity of each site's study population and the lack of a standardised approach across the G6PDd studies conducted in Indonesia. This heterogeneity, its consequent low spatial correlation, and the spatial gaps between the study sites are also reflected in the poor fit of the geospatial model-based map (Appendix p. 30). The Sei Pinang site in Central Kalimantan (n = 347) had the highest G6PDd prevalence (20%) and lowest overall AMM (6·60 U/g Hb, as calculated from reference spectrophotometry measurements; Appendix p. 11). In sites with an anomalously low AMM and deficiency threshold like this, a standardised approach to determining deficiency, such as a point-of-care device with a manufacturer-defined threshold,^55^ may be more suitable.

Previous maps of G6PD variants in Indonesia were published in 2013.^56^ Our updated variant maps include more survey sites and identified a greater number of variants. The maps generated in the 2013 study notably had no variant data available from the provinces of Papua, and only reported 8 out of 15 variants identified in our current analysis. All of the G6PD variants reported in both mapping efforts are classified as Class B, in which carriers are at risk of drug-induced acute haemolytic anaemia.^51^ The variants in Class B were previously classified as Class II and Class III,^52^ which as a whole exhibited a wide range of median and individual G6PD activity levels.^57^ In this study, the Vanua Lava variant was the most frequently reported. Both the genotypic G6PD variant and the phenotypic G6PD activity affect the risk and severity of drug-induced haemolysis, alongside the dose of triggering oxidants, in heterozygous women the proportion of G6PD deficient red blood cells, and possibly the age of the RBC population.^58^ The lack of identifiable G6PD genetic variants in 24% of the genotyped G6PD deficient individuals was likely due to the limitations of the genotyping methods (Appendix pp. 18–20): PCR-based methods could only detect the intended specific variants,^33^^,^^34^^,^^36^^,^^39^^,^^41^^,^^43^, ^44^, ^45^, ^46^, ^47^ and the Sanger sequencing protocol employed by the included studies either performed on specific exons to confirm suspected mutations^34^^,^^36^ or provided incomplete coverage of the gene.^35^^,^^37^^,^^38^^,^^40^^,^^43^, ^44^, ^45^

The current policy for the treatment of uncomplicated *P. vivax* malaria in Indonesia includes a low-daily-dose PQ regimen without prior G6PD testing.^5^ However, this regimen is less efficacious at preventing relapses compared to the intermediate- and high-daily-dose of PQ regimens, with a risk of recurrence of 19% vs 8%.^59^ The prolonged 14-day regimen is also associated with poor adherence and effectiveness (as low as 12% when unsupervised).^60^ Indonesia's future *P. vivax* malaria treatment policies are likely to include the high-daily-dose PQ regimen to facilitate better adherence and efficacy,^3^ but at the increased risk of drug-induced haemolysis^61^ and is only recommended in patients with >70% G6PD activity; the latter requires the support of stringent mandatory quantitative G6PD testing at the point of care.^9^ Our findings underline the need for routine G6PD testing if these novel treatment regimens are to be introduced.

Compared to *P. vivax*-endemic neighbouring countries, routine G6PD testing in Indonesia is less prioritised^8^: Lao PDR and Thailand have implemented country-wide quantitative G6PD testing to guide the use of intermediate-daily-dose PQ or single-dose TQ, respectively, as radical cure, with well-documented successes and challenges,^8^ which may provide insights when Indonesia adapts a high-daily-dose PQ regimen into its treatment policy. On the other hand, radical cure regimens with TQ in Indonesia are not likely to be implemented despite its advantageous single-dose administration, due to the lack of clinically meaningful relapse-free efficacy when TQ was used in combination with DHP, compared to treatment with DHP alone, DHP + low-daily-dose PQ, or DHP + high-daily-dose PQ.^62^^,^^63^

The relevance of routine quantitative G6PD testing to guide high-daily-dose PQ treatment regimen is likely to increase following the increase in reported *P. vivax* malaria cases in Indonesia.^1^ Quantitative point-of-care G6PD diagnostics are costly,^8^ and in Indonesia, the cost-per-test can duplicate or triplicate due to high import duties and additional transport and storage costs, but when combined with better adherence and effectiveness to a short-course PQ regimen, they are expected to reduce the economic burden of *P. vivax* malaria.^10^ Information on local G6PDd prevalence, *P. vivax* malaria incidence, available resources to treat potential haemolytic crisis, available resources for G6PD testing, and cost-benefit analysis will guide policymakers in prioritising areas for implementation, as well as deciding the level of healthcare facilities where G6PD testing will be conducted.

The presented data are subject to a number of limitations. Twenty two percent (19/87) of sites included reported results from less than 100 participants (Appendix pp. 9–17), and the study representativeness criteria include trials, cohort studies, and cross-sectional studies recruiting malaria patients, compromising generalisability. The majority (266/542; 49%) of individuals featured in the variant map came from one province, East Nusa Tenggara. The majority of data from Timika, Papua, were derived from transmigrants from other Indonesian regions rather than indigenous Papuans, which stresses the need to address the paucity of G6PD data from the region. Some studies enrolled only males or females (Tables 1 and 2), and consequently, their reported G6PDd prevalences may differ from the sites' actual population. Overall, studies with an all-female study population had lower G6PDd prevalences (Appendix p. 22), whereas studies with all-male or non-exclusionary study populations showed a significant correlation between G6PDd prevalence and allele frequency (r_s_ = 0·890, Appendix p. 31). Six different qualitative assays were used to screen for G6PDd among the included studies (Appendix pp. 9–17), each with its own deficiency threshold and performance; studies using reference spectrophotometry also employed different kits and instruments, with varying performance and absolute readings.^64^ The model-based map in this study provided predicted mean G6PDd prevalence in areas without G6PD surveys (Fig. 5); however, the uncertainty (95% credible interval) in these areas is notably wide (Appendix p. 29). Furthermore, the predictive model only considered the spatial relationship between the point of prediction and the sites where prevalence was observed, with no other available predictors for G6PDd prevalence (such as ethnicity). Finally, 6 of the included studies were conducted more than 30 years ago and may not represent the current population living in the respective areas.

We present an updated and comprehensive review of available G6PDd data in Indonesia. The prevalence and variants of G6PDd reported vary significantly between sites, but many areas with high *P. vivax* malaria endemicity, most notably the provinces of Papua, lack information on G6PDd altogether, necessitating further collection of local evidence. Information on G6PDd prevalence and variants will provide important evidence for gauging the risks of various PQ regimens for patients with *P. vivax* malaria that support the acceleration of malaria elimination in Indonesia.

## Contributors

AS, IF, IRFE, and AWS provided conceptualisation; AS performed the formal analysis; AS, LVP, IN, and AWS performed data curation; AS, LVP, and AWS accessed and verified the data; LVP, IN, JKB, JH, EK, PCL, SGM, RN, APP, JRP, HDP, RNP, RTP, WS, HS, IS, DS, and LT performed the investigation; BL and AWS provided supervision; AS and AWS wrote the original draft; all authors performed review and editing of the draft. All authors had full access to all the data and had final responsibility for the decision to submit for publication.

## Data sharing statement

This study does not involve collecting primary data; all IPD and aggregate data analysed were obtained from the published and unpublished studies as listed in the results section. Data ownership accordingly remains with each study's respective primary investigators, and any data request should be addressed to them. Aggregate G6PD data used to generate the maps are available in Appendix pp. 9–17, and the corresponding reproducible code is available at https://github.com/arkashas/G6PD-indonesia-map/.

## Editor note

The Lancet Group takes a neutral position with respect to territorial claims in published maps and institutional affiliations.

## Declaration of interests

All authors declare no competing interests.
