# Supplementary material for: G6PD deficiency in Indonesia: a systematic review and update of prevalence and variant maps in the context of malaria elimination
Source: Lancet Reg Health West Pac. 2026 Mar 25;69:101840. doi: 10.1016/j.lanwpc.2026.101840 (PMC13053798; doi:10.1016/j.lanwpc.2026.101840)
Supplement: Supplementary Materials [file mmc1.pdf]

Appendix for “G6PD Deficiency in Indonesia: A Systematic Review and Update of Prevalence and Variant Maps”

Table of Contents

Appendix A. PRISMA Checklist 2
Appendix B. Representativeness, inclusion, and exclusion criteria 4
Appendix C. JBI Critical Appraisal Checklist 5
Appendix D. Supplementary tables 8
Supplementary Table 1. Details of ethical approvals of the included unpublished studies 8
Supplementary Table 2. G6PDd prevalence, G6PDd allele frequency, and prevalence of females with G6PD activity of <70% in Indonesia 9
Supplementary Table 3. G6PD variants reported among individuals classified as G6PD deficient in Indonesia 18
Appendix E. Supplementary Figures 21
Supplementary Figure 1. Forest plot of the prevalences of G6PDd from eligible studies in Indonesia 21
Supplementary Figure 2. Forest plot of subgroup analyses of the estimated prevalence of G6PD Deficiency from eligible studies in Indonesia to investigate potential sources of heterogeneity. 22
Supplementary Figure 3. Forest plot of the G6PDd allele frequencies from eligible studies in Indonesia 23
Supplementary Figure 4. Forest plot of the prevalences of female participants with G6PD activity <70% of normal from eligible studies in Indonesia 24
Supplementary Figure 5. A funnel plot visualising the relationship between site-specific effect sizes (Freeman-Tukey’s p) and their precision (Standard error) of all included studies 25
Supplementary Figure 6. Summary table of the leave-one-out sensitivity analysis of the prevalence of G6PD deficiency from eligible studies in Indonesia (87 sites) 26
Supplementary Figure 7. Summary table of the leave-one-out sensitivity analysis of the G6PD deficiency allele frequencies from eligible studies in Indonesia (82 sites) 27
Supplementary Figure 8. Summary table of the leave-one-out sensitivity analysis of the prevalence of female participants with G6PD activity <70% of normal from eligible studies in Indonesia (35 sites) 28
Supplementary Figure 9. Geostatistical map of lower (A) and upper (B) limits of predicted G6PD prevalence in Indonesia modelled from site-specific G6PDd prevalence data 29
Supplementary Figure 10. Histograms showing the distribution of CPO values (A) and PIT values (B) for each observation in the geostatistical model of G6PDd prevalence 30
Supplementary Figure 11. Scatterplot showing the correlation between G6PDd prevalence and allele frequency among study sites with male participants 31
References 32

Appendix A. PRISMA Checklist.

Section and Topic	Item #	Checklist item	Location where item is reported
**TITLE**			
Title	1	Identify the report as a systematic review.	Page 1
**ABSTRACT**			
Abstract	2	See the PRISMA 2020 for Abstracts checklist.	Pages 2-3
**INTRODUCTION**			
Rationale	3	Describe the rationale for the review in the context of existing knowledge.	Page 7
Objectives	4	Provide an explicit statement of the objective(s) or question(s) the review addresses.	Page 7
**METHODS**			
Eligibility criteria	5	Specify the inclusion and exclusion criteria for the review and how studies were grouped for the syntheses.	Pages 7-8; Appendix p. 4
Information sources	6	Specify all databases, registers, websites, organisations, reference lists and other sources searched or consulted to identify studies. Specify the date when each source was last searched or consulted.	Page 7
Search strategy	7	Present the full search strategies for all databases, registers and websites, including any filters and limits used.	Page 7
Selection process	8	Specify the methods used to decide whether a study met the inclusion criteria of the review, including how many reviewers screened each record and each report retrieved, whether they worked independently, and if applicable, details of automation tools used in the process.	Pages 7-8; Appendix p. 4
Data collection process	9	Specify the methods used to collect data from reports, including how many reviewers collected data from each report, whether they worked independently, any processes for obtaining or confirming data from study investigators, and if applicable, details of automation tools used in the process.	Pages 8-9
Data items	10a	List and define all outcomes for which data were sought. Specify whether all results that were compatible with each outcome domain in each study were sought (e.g. for all measures, time points, analyses), and if not, the methods used to decide which results to collect.	Pages 8-9
	10b	List and define all other variables for which data were sought (e.g. participant and intervention characteristics, funding sources). Describe any assumptions made about any missing or unclear information.	Pages 8-9
Study risk of bias assessment	11	Specify the methods used to assess risk of bias in the included studies, including details of the tool(s) used, how many reviewers assessed each study and whether they worked independently, and if applicable, details of automation tools used in the process.	Page 8
Effect measures	12	Specify for each outcome the effect measure(s) (e.g. risk ratio, mean difference) used in the synthesis or presentation of results.	Page 10
Synthesis methods	13a	Describe the processes used to decide which studies were eligible for each synthesis (e.g. tabulating the study intervention characteristics and comparing against the planned groups for each synthesis (item #5)).	Page 9
	13b	Describe any methods required to prepare the data for presentation or synthesis, such as handling of missing summary statistics, or data conversions.	Page 9
	13c	Describe any methods used to tabulate or visually display results of individual studies and syntheses.	Page 10
	13d	Describe any methods used to synthesize results and provide a rationale for the choice(s). If meta-analysis was performed, describe the model(s), method(s) to identify the presence and extent of statistical heterogeneity, and software package(s) used.	Page 10
	13e	Describe any methods used to explore possible causes of heterogeneity among study results (e.g. subgroup analysis, meta-regression).	Page 10
	13f	Describe any sensitivity analyses conducted to assess robustness of the synthesized results.	Page 10
Reporting bias assessment	14	Describe any methods used to assess risk of bias due to missing results in a synthesis (arising from reporting biases).	Page 8
Certainty assessment	15	Describe any methods used to assess certainty (or confidence) in the body of evidence for an outcome.	N/A
**RESULTS**			
Study selection	16a	Describe the results of the search and selection process, from the number of records identified in the search to the number of studies included in the review, ideally using a flow diagram.	Page 12; Figure 1
	16b	Cite studies that might appear to meet the inclusion criteria, but which were excluded, and explain why they were excluded.	Page 12

Section and Topic	Item #	Checklist item	Location where item is reported
Study characteristics	17	Cite each included study and present its characteristics.	Tables 1 & 2
Risk of bias in studies	18	Present assessments of risk of bias for each included study.	Appendix C
Results of individual studies	19	For all outcomes, present, for each study: (a) summary statistics for each group (where appropriate) and (b) an effect estimate and its precision (e.g. confidence/credible interval), ideally using structured tables or plots.	Appendix pp.9-17, 21, 23-24
Results of syntheses	20a	For each synthesis, briefly summarise the characteristics and risk of bias among contributing studies.	Pages 21-22
	20b	Present results of all statistical syntheses conducted. If meta-analysis was done, present for each the summary estimate and its precision (e.g. confidence/credible interval) and measures of statistical heterogeneity. If comparing groups, describe the direction of the effect.	Pages 21-22; Appendix pp. 21, 23-24
	20c	Present results of all investigations of possible causes of heterogeneity among study results.	Page 21
	20d	Present results of all sensitivity analyses conducted to assess the robustness of the synthesized results.	Page 22
Reporting biases	21	Present assessments of risk of bias due to missing results (arising from reporting biases) for each synthesis assessed.	Page 22
Certainty of evidence	22	Present assessments of certainty (or confidence) in the body of evidence for each outcome assessed.	N/A
**DISCUSSION**			
Discussion	23a	Provide a general interpretation of the results in the context of other evidence.	Pages 25-26
	23b	Discuss any limitations of the evidence included in the review.	Page 28
	23c	Discuss any limitations of the review processes used.	Page 28
	23d	Discuss implications of the results for practice, policy, and future research.	Page 29
**OTHER INFORMATION**			
Registration and protocol	24a	Provide registration information for the review, including register name and registration number, or state that the review was not registered.	Pages 2 & 7
	24b	Indicate where the review protocol can be accessed, or state that a protocol was not prepared.	Pages 2 & 7
	24c	Describe and explain any amendments to information provided at registration or in the protocol.	PROSPERO 2022 CRD42022368319
Support	25	Describe sources of financial or non-financial support for the review, and the role of the funders or sponsors in the review.	Page 30
Competing interests	26	Declare any competing interests of review authors.	Page 29
Availability of data, code and other materials	27	Report which of the following are publicly available and where they can be found: template data collection forms; data extracted from included studies; data used for all analyses; analytic code; any other materials used in the review.	Pages 29-30

Appendix B. Representativeness, inclusion, and exclusion criteria for full-text screening of eligible publications.

For studies using qualitative and quantitative assays:

- Representativeness criteria:
    - Study population residing permanently (>5 years, or since birth for participants aged ≤5 years) on study site
- Inclusion criteria:
    - Study site information available
    - Number of tested participants and number of G6PD deficient participants reported
- Exclusion criteria:
    - Case-control study designs
    - Case reports and case series study designs
    - Purposive sampling method based on ethnicity or blood disorders
    - Meta-analysis studies
    - Duplicate studies (reporting the same results from the same site)
    - Participant age <3 months
    - <35 male participants (for studies using quantitative assay only)

Studies using molecular assays:

- Representativeness criteria:
    - Study population residing permanently (>5 years, or since birth for participants aged ≤5 years) on study site
- Inclusion criteria:
    - Study site information available
- Exclusion criteria:
    - Meta-analysis studies

Appendix C. JBI Critical Appraisal Checklist for studies reporting prevalence data.

Study	Overall appraisal	Was the sample frame appropriate to address the target population?	Were study participants sampled in an appropriate way?	Was the sample size adequate?	Were the study subjects and the setting described in detail?	Was the data analysis conducted with sufficient coverage of the identified sample?	Were valid methods used for the identification of the condition?	Was the condition measured in a standard, reliable way for all participants?	Was there appropriate statistical analysis?	Was the response rate adequate, and if not, was the low response rate managed appropriately?
Eng, 1964[1]	Include	Yes	Yes	Yes	Yes	N/A	Yes	Yes	Yes	N/A
Breguet, 1982[2]	Include	Yes	Yes	Yes	Yes	N/A	Yes	Yes	Yes	N/A
Matsuoka, 1986[3]	Include	Yes	Yes	Yes	Yes	N/A	Yes	Yes	Yes	N/A
Jones, 1990[4]	Include	Yes	Yes	Yes	Yes	N/A	Yes	Yes	Yes	N/A
Fryauff, 1995[5]	Include	Yes	Yes	No	Yes	N/A	Yes	Yes	Yes	N/A
Azhar, 1998[6]	Include	Yes	Yes	Yes	Yes	N/A	Yes	Yes	Yes	N/A
Tantular, 1999[7]	Include	Yes	Yes	Yes	Yes	N/A	Yes	Yes	Yes	N/A
Azhar, 2001[8]	Include	Yes	Yes	Yes	Yes	N/A	Yes	Yes	Yes	N/A
Syahyuni, 2003[9]	Include	Yes	Yes	Yes	Yes	N/A	Yes	Yes	Yes	N/A
Jalloh, 2004[10]	Include	Yes	Yes	Yes	Yes	N/A	Yes	Yes	Yes	N/A
Shimizu, 2005[11]	Include	Yes	Yes	Yes	Yes	N/A	Yes	Yes	Yes	N/A
Lederman, 2006[12]	Include	Yes	Yes	No	Partial	N/A	Yes	Yes	Yes	N/A
Tuda, 2007[13]	Include	Yes	Yes	Yes	Yes	N/A	Yes	Yes	Yes	N/A
Soemantri, 1995[14]	Include	Yes	Yes	Yes	Yes	N/A	Yes	Yes	Yes	N/A
Davy, 2000[15]	Include	Yes	Yes	No	Yes	N/A	Yes	Yes	Yes	N/A
Hardjowasito, 2001[16]	Include	Yes	Yes	No	Partial	N/A	Yes	Yes	Yes	N/A
Iwai, 2001[17]	Include	Yes	Yes	Yes	Yes	N/A	Yes	Yes	Yes	N/A
Matsuoka, 2003[18]	Include	Yes	Yes	Yes	Yes	N/A	Yes	Yes	Yes	N/A
Kawamoto, 2006[19]	Include	Yes	Yes	Yes	Yes	N/A	Yes	Yes	Yes	N/A
Suhartati, 2006[20]	Include	Yes	Yes	Yes	Yes	N/A	Yes	Yes	Yes	N/A
Tantular, 2010[21]	Include	Yes	Yes	Yes	Yes	N/A	Yes	Yes	Yes	N/A
Asih, 2012[22]	Include	Yes	Yes	Yes	Partial	N/A	Yes	Yes	Yes	N/A
Hutagalung, 2015[23]	Include	Yes	Yes	Yes	Yes	N/A	Yes	Yes	Yes	N/A

Satyagraha, 2015[24]	Include	Yes	Yes	Yes	Yes	N/A	Yes	Yes	Yes	N/A
Satyagraha, 2016[25]	Include	Yes	Yes	Yes	Yes	N/A	Yes	Yes	Yes	N/A
Satyagraha, 2021[26]	Include	Yes	Yes	Yes	Yes	N/A	Yes	Yes	Yes	N/A
Sadhewa, 2024a[27]	Include	Yes	Yes	Yes	Yes	N/A	Yes	Yes	Yes	N/A
Sadhewa, 2024b[28]	Include	Yes	Yes	Yes	Yes	N/A	Yes	Yes	Yes	N/A
Banjarmasin & Banjarbaru (Satyagraha, 2012, unpublished)	Include	Yes	Yes	Yes	Yes	N/A	Yes	Yes	Yes	N/A
Maba (Satyagraha, 2012, unpublished)	Include	Yes	Yes	Yes	Yes	N/A	Yes	Yes	Yes	N/A
Bangka (Satyagraha, 2013, unpublished)	Include	Yes	Yes	Yes	Yes	N/A	Yes	Yes	Yes	N/A
PLK-MBS (Syafruddin & Setiadi, 2014, unpublished)	Include	Yes	Yes	Yes	Yes	N/A	Yes	Yes	Yes	N/A
Orang Rimba (Sudoyo, 2015, unpublished)	Include	Yes	Yes	Yes	Yes	N/A	Yes	Yes	Yes	N/A
Mentawai (Sudoyo, 2016, unpublished)	Include	Yes	Yes	No	Yes	N/A	Yes	Yes	Yes	N/A
IMPROV Substudy (Sutanto, Pasaribu, & Satyagraha, 2016, unpublished)	Include	Yes	Yes	Yes	Yes	N/A	Yes	Yes	Yes	N/A
Seram Utara (Sudoyo, 2017, unpublished)	Include	Yes	Yes	No	Yes	N/A	Yes	Yes	Yes	N/A
Enggano (Syafruddin, 2017, unpublished)	Include	Yes	Yes	Yes	Yes	N/A	Yes	Yes	Yes	N/A
Keerom (Syafruddin,	Include	Yes	Yes	Yes	Yes	N/A	Yes	Yes	Yes	N/A

2017, unpublished)										
ELIPI (Poespoprodjo, 2018, unpublished)	Include	Yes	Yes	Yes	Yes	N/A	Yes	Yes	Yes	N/A
ACROSS Boking (Noviyanti, 2018, unpublished)	Include	Yes	Yes	Yes	Yes	N/A	Yes	Yes	Yes	N/A
Satyagraha, 2020 (Timika, unpublished)	Include	Yes	Yes	Yes	Yes	N/A	Yes	Yes	Yes	N/A
**Comment**		**Already part of the systematic search criteria.**	**Already part of the systematic search criteria.**	**The adequate sample size required to estimate G6PDd prevalence with 95% confidence level, 5% margin of error, and expected prevalence of 10% is 139. Studies where the adequate sample size were not met are still included to obtain the most complete picture of G6PDd prevalence in Indonesia.**	**Study location is already part of the systematic search criteria and well-describe in all studies. Some studies did not present aggregated participant sex data and accordingly not included in sex-specific maps (noted as Partial).**	**All subgroups of the identified sample respond at the same rate.**	**Already part of the systematic search criteria.**	**While some studies employed qualitative G6PD assays, all participants within the same study were measured in the same way with well-documented methods.**	**All numerators and denominators of the reported prevalence are clearly reported and were able to be extracted (see S1 Table).**	**Only positive responses (participants with G6PD testing results) were considered.**

Appendix D. Supplementary Tables

Supplementary Table 1. Details of ethical approvals of the included unpublished studies.

Study (Local PIs, Year of Study)	Institutional Ethics Committee	Ethical Approval Number	Approval Date
Banjarmasin & Banjarbaru (Satyagraha, 2012)	Eijkman Institute Research Ethics Commission, Indonesia	Project No. 52	15 June 2012
Maba (Satyagraha, 2012)	Eijkman Institute Research Ethics Commission, Indonesia	Project No. 52	15 June 2012
Bangka (Satyagraha, 2013)	Eijkman Institute Research Ethics Commission, Indonesia	Project No. 59	1 July 2013
PLK-MBS (Syafruddin & Setiadi, 2014)	Eijkman Institute Research Ethics Commission, Indonesia	Project No. 59	1 July 2013
Orang Rimba (Sudoyo, 2015)	Eijkman Institute Research Ethics Commission, Indonesia	Project No. 90	29 October 2015
Mentawai (Sudoyo, 2016)	Eijkman Institute Research Ethics Commission, Indonesia	Project No. 90	29 October 2015
IMPROV Substudy (Sutanto, Pasaribu, & Satyagraha, 2016)	Health Research Ethics Committee, Faculty of Medicine, Universitas Indonesia, Cipto Mangunkusumo Hospital, Indonesia	No. 59/H2.F1/ETIK/2014	27 January 2014
	Human Research Ethics Committee of the Northern Territory Department of Health and Menzies School of Health Research, Australia	2013-1991	26 June 2014 (Protocol v4)
	Oxford Tropical Research Ethics Committee, University of Oxford, UK	1014-13	30 June 2014 (Protocol v4)
	Health Research Ethics Committee, Faculty of Medicine, Universitas Indonesia, Cipto Mangunkusumo Hospital, Indonesia	No. 743/UN2.F1/ETIKlX/2014	6 October 2014
	Health Research Ethics Committee, Faculty of Medicine, Universitas Indonesia, Cipto Mangunkusumo Hospital, Indonesia	No. 289/UN2.F1/ETIK/III/2017	20 March 2017
Seram Utara (Sudoyo, 2017)	Eijkman Institute Research Ethics Commission, Indonesia	Project No. 90	29 October 2015
Enggano (Syafruddin, 2017)	Eijkman Institute Research Ethics Commission, Indonesia	Project No. 111	28 August 2017
Keerom (Syafruddin, 2017)	Research Ethics Comission, Hasanuddin University	No. 663/H4.8.4.5.31/PP36-KOMETIK/2016<br><br>Renewal: No. 356/H4.8.4.5.31/PP36-KOMETIK/2017	26 May 2016<br><br>Renewal: 31 May 2017
	Eijkman Institute Research Ethics Commission, Indonesia	Project No. 111	28 August 2017
ELIPI (Poespoprodjo, 2018)	Medical and Health Research Ethics Committee (MHREC), Faculty of Medicine, Gadjah Mada University, Dr. Sardjito General Hospital, Indonesia	Ref. No. KE/FK/630/EC/2016	10 June 2016
	Human Research Ethics Committee of the Northern Territory Department of Health and Menzies School of Health Research, Australia	2015-2409	21 June 2016
ACROSS Boking (Noviyanti, 2018)	Human Research Ethics Committee of the Northern Territory Department of Health and Menzies School of Health Research, Australia	2017-3010	18 December 2017
	Eijkman Institute Research Ethics Commission, Indonesia	Project No. 121	25 July 2018
ACROSS Timika (Satyagraha, 2020)	Human Research Ethics Committee of the Northern Territory Department of Health and Menzies School of Health Research, Australia	2019-3499	27 November 2019
	Eijkman Institute Research Ethics Commission, Indonesia	Project No. 135	21 November 2019
IGDP (Malik, 2024)	Eijkman Institute Research Ethics Commission, Indonesia	Project No. 90	29 October 2015
	Nanyang Technological University Institutional Review Board, Singapore	IRB-2014-12-011	

Supplementary Table 2. G6PDd prevalence, G6PDd allele frequency, and prevalence of females with G6PD activity of <70% in Indonesia.

Province	City/ Regency	Site	Study	Sample Size	Study Population	Assay Type	Assay	Assay Notes	AMM (U/g Hb)	G6PDd Prevalence (n deficient/n total) [95% CI]	Allele Frequency (n male deficient/n male total) [95% CI]	Prevalence of females with G6PD activity <70% (n female with <70% act./n female total) [95% CI]
Aceh	Sabang City	14 villages in Sabang City	Asih, 2012[22]	937	Population-based survey volunteers	QUAL	WST			0.21% (2/937) [0.03-0.77%]		
Aceh	Banda Aceh	Great Aceh	Azhar, 2001[8]	50	Healthy university and high school students (majority)	QUAL	FST			4.00% (2/50) [0.49-13.71%]	6.25% (2/32) [0.77-20.81%]	
Aceh	Central Aceh	Middle Aceh	Azhar, 2001[8]	89	Healthy university and high school students (majority)	QUAL	FST			1.12% (1/89) [0.03-6.10%]	1.79% (1/56) [0.05-9.55%]	
North Sumatra	Nias Utara	Afia	Matsuoka, 1986[3]	81	Elementary school students	QUAL	MTT/PMS			2.47% (2/81) [0.30-8.64%]	3.77% (2/53) [0.46-12.98%]	
North Sumatra	Nias Selatan	Boto Hilitano	Matsuoka, 1986[3]	118	Elementary school students	QUAL	MTT/PMS			2.54% (3/118) [0.53-7.25%]	5.17% (3/58) [1.08-14.38%]	
North Sumatra	Nias Selatan	Hiliana'a	Matsuoka, 1986[3]	82	Elementary school students	QUAL	MTT/PMS			4.88% (4/82) [1.34-12.02%]	10.00% (4/40) [2.79-23.66%]	
North Sumatra	Medan	Medan	Matsuoka, 1986[3]	260	Elementary school students	QUAL	MTT/PMS			3.18% (9/283) [1.46-5.95%]	6.77% (9/133) [3.14-12.46%]	
			Davy, 2000[15]	23	Healthy male participants		FST					
North Sumatra	Batubara	Durian	Matsuoka, 1986[3]	73	Elementary school students	QUAL	MTT/PMS			1.37% (1/73) [0.03-7.40%]	2.70% (1/37) [0.07-14.16%]	
North Sumatra	Batubara	Perupuk & Guntung	Matsuoka, 1986[3]	533	Elementary school students	QUAL	MTT/PMS			2.25% (12/533) [1.17-3.90%]	4.07% (11/270) [2.05-7.17%]	
North Sumatra	Labuhanbatu Utara	Tanjung Leidong	IMPROV Substudy (Sutanto, Pasaribu, & Satyagraha, 2016, unpublished)	308	Individuals with fever/history of fever, visiting	QUANT	SPECTRO	Trinity Kit	8.63	0.65% (2/308) [0.08-2.33%]	1.47% (1/68) [0.04-7.92%]	2.92% (7/240) [1.18-5.92%]

					community health centres							
West Sumatra	Kepulauan Mentawai	Mentawai	Mentawai (Sudoyo, 2016, unpublished)	94	Population-based survey, 1 individual per household	QUANT	SPECTRO	Trinity Kit	7.13	0.00% (0/94) [0.00-3.85%]	0.00% (0/94) [0.00-3.85%]	
Jambi	Batang Hari	Bukit Dua Belas National Park	Orang Rimba (Sudoyo, 2015, unpublished)	239	Population-based survey, 1 individual per household	QUANT	SPECTRO	Trinity Kit	8.21	0.00% (0/239) [0.00-1.53%]	0.00% (0/111) [0.00-3.27%]	0.78% (1/128) [0.02-4.28%]
Bengkulu	Bengkulu Utara	Arga Makmur	Enggano (Syafruddin, 2017, unpublished)	328	Population-based survey volunteers (residents)	QUANT	SPECTRO	Trinity Kit	8.41	0.30% (1/328) [0.01-1.69%]	0.00% (0/86) [0.00-4.20%]	4.13% (10/242) [2.00-7.47%]
Bengkulu	Bengkulu Utara	Enggano	Enggano (Syafruddin, 2017, unpublished)	155	Population-based survey volunteers (residents)	QUANT	SPECTRO	Trinity Kit	8.63	4.52% (7/155) [1.83-9.08%]	0.00% (0/63) [0.00-5.69%]	7.61% (7/92) [3.11-15.05%]
Bangka Belitung Islands	Bangka	Bangka	Davy, 2000[15]	44	Healthy male participants	QUAL	FST			2.72% (10/368) [1.31-4.94%]	6.06% (10/165) [2.94-10.86%]	5.42% (11/203) [2.74-9.49%]
			Bangka (Satyagraha, 2013, unpublished)	324	Healthy population-survey volunteers (residents) aged >6 years old	QUANT	SPECTRO	Trinity Kit	8.90			
Bangka Belitung Islands	Bangka Tengah	Bangka Tengah	Bangka (Satyagraha, 2013, unpublished)	282	Healthy population-survey volunteers (residents) aged >6 years old	QUANT	SPECTRO	Trinity Kit	9.69	1.06% (3/282) [0.22-3.08%]	2.88% (3/104) [0.60-8.20%]	2.25% (4/178) [0.62-5.65%]
Lampung	Pesawaran	Hanura	IMPROV Substudy (Sutanto, Pasaribu, & Satyagraha, 2016, unpublished)	300	Individuals with fever/history of fever, visiting community health centres	QUANT	SPECTRO	Trinity Kit	7.35	7.00% (21/300) [4.39-10.50%]	13.91% (16/115) [8.17-21.61%]	14.59% (27/185) [9.84-20.52%]
DKI Jakarta	Jakarta Pusat	Jakarta	Eng, 1964[1]	446	Healthy participants and hospital patients	QUAL	BCB; MRT			1.12% (5/446) [0.36-2.60%]	1.12% (5/446) [0.36-2.60%]	
Central Java	Purworejo	Menoreh Hills	Lederman, 2006[12]	124	Participants with uncomplicate	QUAL	FST			0.81% (1/124) [0.02-4.41%]		

					d *P. falciparum* malaria							
Central Java	Semarang	Semarang	Soemantri, 1995[14]	169	Adult male participants	QUAL	ELE			13.61% (23/169) [8.83-19.72%]	13.61% (23/169) [8.83-19.72%]	
East Java	Surabaya	Surabaya	Jalloh, 2004[10]	307	Survey volunteers	QUAL	WST			1.63% (5/307) [0.53-3.76%]	2.86% (4/140) [0.78-7.15%]	
Bali	Karangasem	Tenganan Pageringsingan	Breguet, 1982[2]	316	Residents aged >12 years	QUAL	ELE			5.06% (16/316) [2.92-8.09%]	9.64% (16/166) [5.61-15.18%]	
Central Kalimantan	Kotawaringin Timur	Waringin Agung	PLK-MBS (Syafruddin & Setiadi, 2014, unpublished)	293	Population-based survey volunteers (residents)	QUANT	SPECTRO	Trinity Kit	8.69	18.77% (55/293) [14.47-23.72%]	20.95% (31/148) [14.70-28.39%]	24.83% (36/145) [18.03-32.68%]
Central Kalimantan	Gunung Mas	Gunung Mas	PLK-MBS (Syafruddin & Setiadi, 2014, unpublished)	230	Population-based survey volunteers (residents)	QUANT	SPECTRO	Trinity Kit	7.85	3.91% (9/230) [1.80-7.30%]	5.52% (8/145) [2.41-10.58%]	32.94% (28/85) [23.13-43.98%]
Central Kalimantan	Palangkaraya	Palangkaraya[15]	Davy, 2000[15]	50	Healthy male participants	QUAL	FST			6.00% (3/50) [1.25-16.55%]	6.00% (3/50) [1.25-16.55%]	
Central Kalimantan	Kapuas	Sei Pinang	PLK-MBS (Syafruddin & Setiadi, 2014, unpublished)	347	Population-based survey volunteers (residents)	QUANT	SPECTRO	Trinity Kit	6.60	19.88% (69/347) [15.81-24.48%]	25.85% (38/147) [18.99-33.71%]	36.50% (73/200) [29.35-43.07%]
Central Kalimantan	Murung Raya	Murung Raya	PLK-MBS (Syafruddin & Setiadi, 2014, unpublished)	146	Population-based survey volunteers (residents)	QUANT	SPECTRO	Trinity Kit	8.80	6.16% (9/146) [2.86-11.38%]	13.04% (9/69) [6.14-23.32%]	11.69% (9/77) [5.49-21.03%]
Central Kalimantan	Kapuas	Pujon	PLK-MBS (Syafruddin & Setiadi, 2014, unpublished)	183	Population-based survey volunteers (residents)	QUANT	SPECTRO	Trinity Kit	11.20	4.37% (8/183) [1.91-8.43%]	5.13% (6/117) [1.90-10.83%]	15.15% (10/66) [7.51-26.10%]
Central Kalimantan	Barito Utara	Barito Utara	PLK-MBS (Syafruddin & Setiadi, 2014, unpublished)	331	Population-based survey volunteers (residents)	QUANT	SPECTRO	Trinity Kit	8.48	10.57% (35/331) [7.48-14.40%]	5.02% (11/219) [2.53-8.81%]	44.64% (50/112) [35.24%-54.33%]
South Kalimantan	Banjarmasin City & Banjarbaru City	2 cities in South Kalimantan	Banjarmasin & Banjarbaru (Satyagraha, 2012, unpublished)	201	Healthy vocational school students (residents)	QUANT	SPECTRO	Trinity Kit	7.18	1.49% (3/201) [0.31-4.30%]	1.30% (1/77) [0.03-7.02%]	20.97% (26/124) [14.18-29.19%]
North Kalimantan	Malinau	Malinau	Sadhewa, 2024a[27]	145	Individuals aged ≥6 years visiting	QUANT	SPECTRO	Pointe Kit	11.12	0.69% (1/145) [0.02-3.78%]	0.00% (0/45) [0.00-7.87%]	4.00% (4/100) [1.10-9.93%]

			Sadhewa, 2024b[28]		community health centres							
Southeast Sulawesi	Konawe	Lambuya Village	Tantular, 2010[21]	77	Population-based survey volunteers	QUAL	WST	Modified with less dye mixture		1.30% (1/77) [0.03-7.02%]	2.22% (1/45) [0.06-11.77%]	
Southeast Sulawesi	Muna	2 villages in Muna Island	Tantular, 2010[21]	122	Population-based survey volunteers	QUAL	WST	Modified with less dye mixture		1.64% (2/122) [0.20-5.80%]	2.82% (2/71) [0.34-9.81%]	
North Sulawesi	Bolaang-Mongondow	2 Elementary Schools in Bolaang-Mongondow Regency	Tuda, 2007[13]	168	Elementary school students	QUAL	WST	Dojindo Kit		3.57% (6/168) [1.32-7.61%	6.74% (6/89) [2.51-14.10%	
North Sulawesi	Minahasa	5 villages in Minahasa Regency	Tantular, 2010[21]	319	Population-based survey volunteers	QUAL	WST	Modified with less dye mixture		0.63% (2/319) [0.08-2.25%]	1.27% (2/158) [0.15-4.50%]	
North Sulawesi	Minahasa Utara	2 Elementary Schools in Minahasa Utara Regency	Tuda, 2007[13]	195	Elementary school students	QUAL	WST	Dojindo Kit		5.13% (10/195) [2.49-9.23%]	9.62% (10/104) [4.71-16.97%]	
North Sulawesi	Bitung City	1 Elementary school in Bitung City	Tuda, 2007[13]	79	Elementary school students	QUAL	WST	Dojindo Kit		0.00% (0/79) [0.00-4.56%]	0.00% (0/50) [0.00-7.11%]	
North Sulawesi	Minahasa Utara	3 villages in Bangka Island	Tantular, 2010[21]	32	Population-based survey volunteers	QUAL	WST	Modified with less dye mixture		12.50% (4/32) [3.51-28.99%]	18.18% (4/22) [5.19-40.28%]	
West Nusa Tenggara	Sumbawa	Sumbawa	Azhar, 1998[6]	114	High school students (majority) and healthy adults	QUAL	NIR			6.14% (7/114) [2.50-12.24%]	5.00% (2/40) [0.61-16.92%]	
East Nusa Tenggara	Sumba Barat Daya	Kodi	Satyagraha, 2015[24]	331	Population-based survey volunteers (residents)	QUANT	SPECTRO	Trinity Kit	10.34	9.06% (30/331) [6.20-12.69%]	16.78% (24/143) [11.06-23.94%]	16.49% (31/188) [11.49-22.58%]
East Nusa Tenggara	Sumba Barat Daya	Kodi Balaghar[26]	Satyagraha, 2021[26]	1350	Female healthy volunteers	QUANT	SPECTRO	Trinity Kit	10.89	1.26% (17/1350) [0.74-2.01%]		11.85% (160/1350) [10.18-13.70%]
East Nusa Tenggara	Sumba Barat Daya	Umbu Ngedo[26]	Satyagraha, 2021[26]	678	Female healthy volunteers	QUANT	SPECTRO	Trinity Kit	11.75	2.06% (14/678) [1.13-3.44%]		13.13% (89/678) [10.68-15.90%]
East Nusa Tenggara	Sumba Barat Daya	Panenggo Ede	Satyagraha, 2016[25]	607	Population-based survey volunteers (residents)	QUANT	SPECTRO	Trinity Kit	9.28	4.94% (30/607) [3.36-6.98%]	9.27% (24/259) [6.03-13.47%]	10.92% (38/348) [7.84-14.68%]
East Nusa Tenggara	Sumba Barat	Lamboya	Satyagraha, 2015[24]	316	Population-based survey	QUANT	SPECTRO	Trinity Kit	10.46	6.65% (21/316) [4.16-9.98%]	12.20% (15/123) [6.99-19.32%]	15.03% (29/193) [9.43-19.70%]

					volunteers (residents)							
East Nusa Tenggara	Sumba Barat Daya	Mata Pyawu	Satyagraha, 2015[24]	152	Population-based survey volunteers (residents)	QUANT	SPECTRO	Trinity Kit	10.84	1.32% (2/152) [0.16-4.67%]	1.69% (1/59) [0.04-9.09%]	2.15% (2/93) [0.26-7.55%]
East Nusa Tenggara	Sumba Barat Daya	Mali Mada	Satyagraha, 2015[24]	171	Population-based survey volunteers (residents)	QUANT	SPECTRO	Trinity Kit	10.20	4.68% (8/171) [2.04-9.01%]	6.67% (5/75) [2.20-14.88%]	7.29% (7/96) [2.98-14.45%]
East Nusa Tenggara	Sumba Barat	Wanokaka	Satyagraha, 2015[24]	358	Population-based survey volunteers (residents)	QUANT	SPECTRO	Trinity Kit	9.69	1.68% (6/358) [0.62-3.61%]	3.87% (6/155) [1.43-8.23%]	4.43% (9/203) [2.05-8.25%]
East Nusa Tenggara	Sumba Tengah	Anakalang	Satyagraha, 2015[24]	163	Population-based survey volunteers (residents)	QUANT	SPECTRO	Trinity Kit	9.29	3.07% (5/163) [1.00-7.01%]	3.28% (2/61) [0.40-11.35%]	5.88% (6/102) [2.19-12.36%]
East Nusa Tenggara	Sumba Tengah	Wairasa	Satyagraha, 2015[24]	185	Population-based survey volunteers (residents)	QUANT	SPECTRO	Trinity Kit	9.47	1.62% (3/185) [0.34-4.67%]	1.52% (1/66) [0.04-8.16%]	5.04% (6/119) [1.87-10.65%]
East Nusa Tenggara	Sumba Tengah	Umbu Ratu Nggay	Satyagraha, 2015[24]	320	Population-based survey volunteers (residents)	QUANT	SPECTRO	Trinity Kit	9.83	2.50% (8/320) [1.09-4.87%]	3.97% (6/151) [1.47-8.45%]	4.73% (8/169) [2.07-9.11%]
East Nusa Tenggara	Manggarai Barat	4 villages near Labuan Bajo & Lembor	Tantular, 2010[21]	228	Population-based survey volunteers	QUAL	WST	Modified with less dye mixture		0.44% (1/228) [0.01%-2.42%]	0.63% (1/159) [0.02-3.45%]	
East Nusa Tenggara	Sumba Timur	3 villages near Waingapu	Azhar, 1998[6]	112	High school students (majority) and healthy adults	QUAL	NIR;[6] WST[21]	Modified with less dye mixture		4.74% (42/886) [3.44-6.35%]	4.94% (31/628) [3.38-6.93%]	
			Tantular, 2010[21]	774	Population-based survey volunteers							
East Nusa Tenggara	Sumba Timur	3 districts in East Sumba Regency	Syahyuni, 2003[9]	210	Grade IV and V elementary school students	QUAL	MTT/PMS			8.57% (36/420) [6.08-11.67%]	8.29% (16/193) [4.81-13.11%]	
			Shimizu, 2005[11]	210	Healthy participants							
East Nusa Tenggara	Ngada	Tiworiwu Village	Tantular, 2010[21]	104	Population-based survey volunteers	QUAL	WST	Modified with less dye mixture		2.88% (3/104) [0.60-8.20%]	4.17% (3/72) [0.87-11.70%]	

East Nusa Tenggara	Ngada	Reo Village	Tantular, 2010[21]	102	Population-based survey volunteers	QUAL	WST	Modified with less dye mixture		7.84% (8/102) [3.45-14.87%]	11.11% (6/54) [4.19-22.63%]	
East Nusa Tenggara	Nagekeo	Tonggo Village	Tantular, 2010[21]	90	Population-based survey volunteers	QUAL	WST	Modified with less dye mixture		1.11% (1/90) [0.03-6.04%]	2.04% (1/49) [0.05-10.85%]	
East Nusa Tenggara	Ende	Ende	Kawamoto, 2006[19]	363	Febrile volunteers	QUAL	WST			4.41% (16/363) [2.54-7.06%]	3.95% (7/177) [1.60-7.98%]	
East Nusa Tenggara	Sikka	Reruwairere Village	Tantular, 2010[21]	225	Population-based survey volunteers	QUAL	WST	Modified with less dye mixture		0.44% (1/225) [0.01-2.45%]	1.20% (1/83) [0.03-6.53%]	
East Nusa Tenggara	Sikka	Maumere	Kawamoto, 2006[19]	745	Febrile volunteers	QUAL	WST			5.23% (39/745) [3.75-7.09%]	6.67% (31/465) [4.57-9.33%]	
East Nusa Tenggara	Sikka	Sikka	Jalloh, 2004[10]	979	Survey volunteers; elementary school students and teachers	QUAL	WST			2.55% (25/979) [1.66-3.75%]	4.13% (21/508) [2.58-6.25%]	
East Nusa Tenggara	Sikka	Maumere & Talibura	Matsuoka, 2003[18]	363	Elementary school students	QUAL	WST			4.41% (16/363) [2.54-7.06%	6.21% (11/177) [3.14-10.85%]	
East Nusa Tenggara	Sikka	Pruda Village	Tantular, 2010[21]	251	Population-based survey volunteers	QUAL	WST	Modified with less dye mixture		7.57% (19/251) [4.62-11.57%]	13.64% (18/132) [8.29-20.69%]	
East Nusa Tenggara	Flores Timur	3 villages in Larantuka	Tantular, 2010[21]	257	Population-based survey volunteers	QUAL	WST	Modified with less dye mixture		0.78% (2/257) [0.09-2.78%]	1.37% (2/146) [0.17-4.86%]	
East Nusa Tenggara	Timor Tengah Selatan	Batu Putih	Hutagalung, 2015[23]	119	Population-based survey volunteers	QUANT	SPECTRO	Randox Kit	12.51	2.52% (3/119) [0.52-7.19%]	3.28% (2/61) [0.40-11.35%]	18.97% (11/58) [9.87-31.41%]
East Nusa Tenggara	Timor Tengah Selatan	3 villages near Soe & Oebobo	Tantular, 2010[21]	196	Population-based survey volunteers	QUAL	WST	Modified with less dye mixture		2.04% (4/196) [0.56-5.14%]	3.19% (3/94) [0.66-9.04%]	
East Nusa Tenggara	Timor Tengah Selatan	Oenino	Hutagalung, 2015[23]	134	Population-based survey volunteers	QUANT	SPECTRO	Randox Kit	9.51	1.49% (2/134) [0.18-5.29%]	2.27% (1/44) [0.06-12.02%]	7.78% (7/90) [3.18-15.37%]
East Nusa Tenggara	Alor	Alor	Azhar, 1998[6]	122	High school students (majority) and healthy adults	QUAL	NIR			6.56% (8/122) [2.87-12.51%]	6.67% (4/60) [1.85-16.20%]	
East Nusa Tenggara	Timor Tengah Selatan	Oe'ekam	Hutagalung, 2015[23]	100	Population-based survey volunteers	QUANT	SPECTRO	Randox Kit	9.40	1.00% (1/100) [0.03-5.45%]	0.00% (0/42) [0.00-8.41%]	6.90% (4/58) [1.91-16.73%]

East Nusa Tenggara	Timor Tengah Selatan	Panite	Hutagalung, 2015[23]	99	Population-based survey volunteers	QUANT	SPECTRO	Randox Kit	8.86	6.06% (6/99) [2.26-12.73%]	8.89% (4/45) [2.48-21.22%]	11.11% (6/54) [4.19-22.63%]
East Nusa Tenggara	Timor Tengah Selatan	Oinlasi	Hutagalung, 2015[23]	100	Population-based survey volunteers	QUANT	SPECTRO	Randox Kit	7.73	1.00% (1/100) [0.03-5.45%]	0.00% (0/35) [0.00-10.00%]	27.69% (18/65) [17.31-40.19%]
East Nusa Tenggara	Timor Tengah Utara	Insana	Hardjowasito, 2001[16]	118	Randomly selected participants	QUAL	MTT/PMS			2.54% (3/118) [0.53-7.25%]		
East Nusa Tenggara	Timor Tengah Selatan	Boking	ACROSS Boking (Noviyanti, 2018, unpublished)	294	Population-based survey, 1 individual per household	QUANT	SPECTRO	Pointe Kit	9.50	1.02% (3/294) [0.21-2.95%]	1.21% (2/165) [0.15-4.31%]	5.43% (7/129) [2.21-10.86%]
North Maluku	Tidore Kepulauan	Siokona	Tantular, 1999[7]	93	Survey volunteers	QUAL	MTT/PMS			1.08% (1/93) [0.03-5.85%]	2.44% (1/41) [0.06-12.86%]	
North Maluku	Tidore Kepulauan	Oba	Tantular, 1999[7]	862	Survey volunteers	QUAL	MTT/PMS			3.48% (30/862) [2.36-4.93%]	5.14% (22/428) [3.25-7.68%]	
North Maluku	Tidore Kepulauan	Oba Selatan	Tantular, 1999[7]	171	Survey volunteers	QUAL	MTT/PMS			6.43% (11/171) [3.25-11.22%]	9.41% (8/85) [4.15-17.71%]	
North Maluku	Halmahera Timur	Maba	Maba (Satyagraha, 2012, unpublished)	140	Healthy middle school students (residents)	QUANT	SPECTRO	Trinity Kit	8.88	1.43% (2/140) [0.17-5.07%]	3.08% (2/65) [0.37-10.68%]	2.67% (2/75) [0.32-9.30%]
Maluku; North Maluku	N/A	Buru & Halmahera	Iwai, 2001[17]	696	Population-based survey volunteers	QUAL	MTT/PMS			6.03% (42/696) [4.38-8.07%]	6.03% (42/696) [4.38-8.07%]	
Maluku	Maluku Barat Daya	Pulau Romang	Suhartati, 2006[20]	64	Visitors of a free community-service health clinic	QUAL	MTT/PMS			3.13% (2/64) [0.38-10.84%]	5.00% (2/40) [0.61-16.92%]	
Maluku	Maluku Barat Daya	Pulau Babar	Suhartati, 2006[20]	58	Visitors of a free community-service health clinic	QUAL	MTT/PMS			5.17% (3/58) [1.08-14.38%]	11.11% (3/27) [2.35-29.16%]	
Maluku	Maluku Tengah	Seram Utara	Seram Utara (Sudoyo, 2017, unpublished)	55	Population-based survey, 1 individual per household	QUANT	SPECTRO	Trinity Kit	10.07	0.00% (0/55) [0.00-6.49%]	0.00% (0/55) [0.00-6.49%]	
Maluku	Kepulauan Tanimbar	Saumlaki	Suhartati, 2006[20]	53	Visitors of a free community-service health clinic	QUAL	MTT/PMS			3.77% (2/53) [0.46-12.98%]	3.85% (1/26) [0.10-19.64%]	

Maluku	Kepulauan Tanimbar	Larat	Suhartati, 2006[20]	59	Visitors of a free community-service health clinic	QUAL	MTT/PMS			6.78% (4/59) [1.88-16.46%]	3.23% (1/31) [0.08-16.70%]	
Maluku	Kota Tual	Pulau Kur	Suhartati, 2006[20]	64	Visitors of a free community-service health clinic	QUAL	MTT/PMS			1.56% (1/64) [0.04-8.40%]	5.00% (1/20) [0.13-24.87%]	
Papua Tengah	Mimika	Timika	ELIPI (Poespoprodjo, 2018, unpublished)	356	Population-based survey volunteers (residents)	QUANT	SPECTRO	Trinity Kit	11.87	1.84% (12/651) [0.96-3.20%]	3.47% (11/317) [1.74-6.12%]	7.49% (25/334) [4.90-10.85%]
			ACROSS Timika (Satyagraha, 2020, unpublished)	295	Individuals with fever/history of fever, visiting Timika Jaya community health centre			Pointe Kit	9.70			
Papua	Keerom	Arso PIR	Jones, 1990[4]	223	Residents, including transmigrants from Java and native Papuan	QUANT	SPECTRO	Sigma Kit		2.69% (6/223) [0.99-5.76%]	2.23% (4/179) [0.61-5.62%]	
Papua	Keerom	Arso XI	Fryauff, 1995[5]	131	Transmigrant residents from Java aged >15 years	QUAL	FST			1.53% (2/131) [0.19-5.41%]	1.53% (2/131) [0.19-5.41%]	
Papua	Keerom	Waris	Keerom (Syafruddin, 2017, unpublished)	206	Population-survey (residents)	QUANT	SPECTRO	Trinity Kit	10.32	4.37% (9/206) [2.02-8.13%]	8.93% (5/56) [2.96-19.62%]	8.67% (13/150) [4.70-14.36%]

NIR = no information retrieved
QUAL = Qualitative assay
QUANT = Quantitative assay
WST = WST-8 1-methoxy PMS-based colorimetric assay
FST = fluorescent spot test
MTT/PMS = any other test with colour-based indicator of activity
SPECTRO = quantitative spectrophotometry
BCB = brilliant cresyl blue / Motulsky's test
MRT = methaemoglobin reduction test
ELE = determination of G6PD variant by electrophoresis

Trinity Kit = G-6-PDH Quantitative kit (Cat. No. 345-B; Trinity Biotech, Ireland)
Pointe Kit = Pointe Scientific G6PD Reagents (Pointe Scientific, USA)
Dojindo Kit = Glucose-6-Phosphate Dehydrogenase Activity Assay Kit (Dojindo, Japan)
Randox Kit = G6PDH assay (Randox Laboratories, UK)
Sigma Kit = Glucose-6-Phosphate Dehydrogenase Activity Assay Kit (Sigma, USA)
AMM = Adjusted male median

Supplementary Table 3. G6PD variants reported among individuals classified as G6PD deficient in Indonesia.

Province	City/Regency	Sites	Study	Genotyping Method	n Genotyped	G6PD Variant	Participants with no identified G6PD Variant
Aceh	Sabang City	14 villages in Sabang City	Asih, 2012[22]	RFLP	2	No known variants detected	2
North Sumatra	Labuhanbatu Utara	Tanjung Leidong	IMPROV Substudy (Sutanto, Pasaribu, & Satyagraha, 2016, unpublished)	SEQ; RFLP	2	1 Mahidol<br>1 Viangchan	0
Bengkulu	Bengkulu Utara	Arga Makmur; Enggano	Enggano (Syafruddin, 2017, unpublished)	SEQ; RFLP	8	3 Chatham<br>3 Vanua Lava<br>2 Viangchan	0
Bangka Belitung Islands	Bangka	Bangka	Davy, 2000[15]<br>Bangka (Satyagraha, 2013, unpublished)	SEQ; PCR; RFLP<br>SEQ	7	2 Canton<br>1 Chatham<br>1 Vanua Lava<br>3 Viangchan	0
Bangka Belitung Islands	Bangka Tengah	Bangka Tengah	Bangka (Satyagraha, 2013, unpublished)	SEQ	3	2 Murcia	1
Lampung	Pesawaran	Hanura	IMPROV Substudy (Sutanto, Pasaribu, & Satyagraha, 2016, unpublished)	SEQ; RFLP	3	1 Chatham<br>1 Coimbra<br>1 Viangchan	0
Central Java	Semarang City	Semarang	Soemantri, 1995[14]	RFLP	16	3 Canton<br>2 Mahidol<br>5 Mediterranean	6
Central Kalimantan	Kotawaringin Timur	Waringin Agung	PLK-MBS (Syafruddin & Setiadi, 2014, unpublished)	SEQ; RFLP	51	9 Vanua Lava<br>6 Viangchan	36
Central Kalimantan	Gunung Mas	Gunung Mas	PLK-MBS (Syafruddin & Setiadi, 2014, unpublished)	RFLP	5	1 Vanua Lava<br>4 Viangchan	0
Central Kalimantan	Palangkaraya	Palangkaraya[15]	Davy, 2000[15]	SEQ; PCR; RFLP	3	1 Viangchan	2
Central Kalimantan	Kapuas	Sei Pinang	PLK-MBS (Syafruddin & Setiadi, 2014, unpublished)	SEQ; RFLP	61	1 Andalus<br>2 Coimbra<br>2 Mahidol<br>1 Nilgiri<br>26 Vanua Lava<br>3 Viangchan	26
Central Kalimantan	Murung Raya	Murung Raya	PLK-MBS (Syafruddin & Setiadi, 2014, unpublished)	RFLP	9	6 Vanua Lava<br>3 Viangchan	0
Central Kalimantan	Barito Utara	Barito Utara	PLK-MBS (Syafruddin & Setiadi, 2014, unpublished)	SEQ; RFLP	25	5 Vanua Lava<br>5 Viangchan	15
South Kalimantan	Banjarmasin City & Banjarbaru City	2 cities in South Kalimantan	Banjarmasin & Banjarbaru (Satyagraha, 2012, unpublished)	SEQ	3	1 Mahidol<br>1 Vanua Lava<br>1 Viangchan	0
North Kalimantan	Malinau	Malinau	Sadhewa, 2024a[27]<br>Sadhewa, 2024b[28]	RFLP	1	1 Viangchan	0
Southeast Sulawesi	Konawe	Lambuya Village	Tantular, 2010[21]	SEQ	1	1 Vanua Lava	0

Southeast Sulawesi	Muna	2 villages in Muna Island	Tantular, 2010[21]	SEQ	2	2 Vanua Lava	0
North Sulawesi	Minahasa	5 villages in Minahasa Regency	Tantular, 2010[21]	SEQ	2	2 Vanua Lava	0
North Sulawesi	Minahasa Utara	3 villages in Bangka Island	Tantular, 2010[21]	SEQ	4	3 Vanua Lava<br>1 Viangchan	0
East Nusa Tenggara	Sumba Barat Daya	Panenggo Ede; Umbu Ngedo; Mali Mada; Mata Pyawu; Kodi	Satyagraha, 2015[24]<br>Satyagraha, 2016[25]<br>Satyagraha, 2021[26]	SEQ; RFLP	89	5 Chatham<br>6 Coimbra<br>46 Vanua Lava<br>30 Viangchan	2
East Nusa Tenggara	Sumba Barat	Lamboya; Wanokaka	Satyagraha, 2015[24]	SEQ	20	9 Chatham<br>1 Kaiping<br>4 Vanua Lava<br>5 Viangchan	1
East Nusa Tenggara	Sumba Tengah	Padira Tana; Wairasa; Anakalang	Satyagraha, 2015[24]	SEQ	12	8 Vanua Lava<br>2 Viangchan	2
East Nusa Tenggara	Sumba Timur	3 villages near Waingapu	Tantular, 2010[21]	SEQ	36	2 Chatham<br>1 Kaiping<br>27 Vanua Lava<br>6 Viangchan	0
East Nusa Tenggara	Ngada	Tiworiwu Village & Reo Village	Tantular, 2010[21]	SEQ	11	1 Coimbra<br>2 Kaiping<br>2 Mediterranean<br>3 Vanua Lava<br>3 Viangchan	0
East Nusa Tenggara	Nagekeo	Tonggo Village	Tantular, 2010[21]	SEQ	1	1 Coimbra	0
East Nusa Tenggara	Ende	Ende	Kawamoto, 2006[19]	SEQ	14	1 Chatham<br>2 Chinese-5<br>2 Kaiping<br>9 Vanua Lava	0
East Nusa Tenggara	Sikka	Maumere; Talibura; Reruwairere Village; Pruda Village	Matsuoka, 2003[18]<br>Kawamoto, 2006[19]<br>Tantular, 2010[21]	SEQ	71	3 Bajo-Maumere<br>16 Chatham<br>10 Coimbra<br>24 Kaiping<br>6 Vanua Lava<br>12 Viangchan	0
East Nusa Tenggara	Flores Timur	3 villages in Larantuka	Tantular, 2010[21]	SEQ	2	1 Vanua Lava<br>1 Viangchan	0
East Nusa Tenggara	Timor Tengah Selatan	Boking; 3 villages near Soe & Oebobo	ACROSS Boking (Noviyanti, 2018, unpublished)<br>Tantular, 2010[21]	SEQ; RFLP<br>SEQ	7	1 Kaiping<br>6 Vanua Lava	0
East Nusa Tenggara	Timor Tengah Utara	Insana	Hardjowasito, 2001[16]	SEQ; PCR	3	1 Coimbra<br>1 Vanua Lava	1
North Maluku	Halmahera Timur	Maba	Maba (Satyagraha, 2012, unpublished)	SEQ	2	2 Vanua Lava	0
Maluku & North Maluku	N/A	Buru & Halmahera	Iwai, 2001[17]	SEQ; PCR; RFLP	42	11 Vanua Lava	31

Maluku	Maluku Barat Daya	Pulau Babar; Pulau Romang	Suhartati, 2006[20]	PCR	5	2 Chatham<br>1 Kaiping<br>1 Vanua Lava	1
Maluku	Kepulauan Tanimbar	Larat; Saumlaki	Suhartati, 2006[20]	PCR	6	5 Vanua Lava	1
Maluku	Kota Tual	Pulau Kur	Suhartati, 2006[20]	PCR	1	No known variants detected	1
Papua	Mimika	Timika	ELIPI (Poespoprodjo, 2018, unpublished)<br>ACROSS Timika (Satyagraha, 2020, unpublished)	SEQ; RFLP	3	1 Chatham<br>2 Vanua Lava	0
Papua	Keerom	Waris	Keerom (Syafruddin, 2017, unpublished)	SEQ; RFLP	9	1 Union<br>7 Vanua Lava	1

RFLP = variant-specific genotyping by restriction fragment length polymorphism
SEQ = any DNA sequencing method
PCR = any type of PCR-based assay besides PCR-RFLP

Appendix E. Supplementary Figures

Supplementary Figure 1. Forest plot of the prevalence of G6PD deficiency from eligible studies in Indonesia. Site identified the site name, and the Appendix reference number or local PI of the study where the data came from.

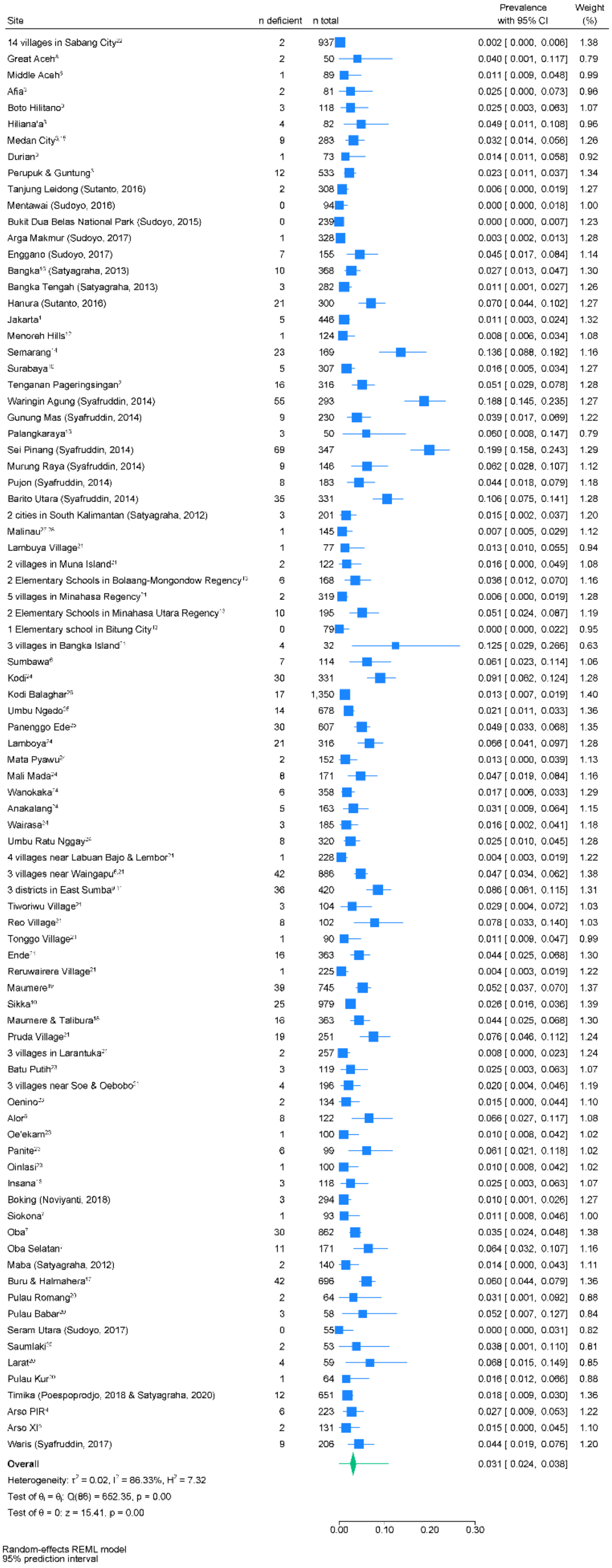

Supplementary Figure 2. Forest plot of subgroup analyses of the estimated prevalence of G6PD deficiency from eligible studies in Indonesia to investigate potential sources of heterogeneity. Three sites[Lederman, Hardjowasito, Asih] were excluded due to lack of sex-disaggregated data. *Study data came from adjacent sites in more than one province [Iwai].

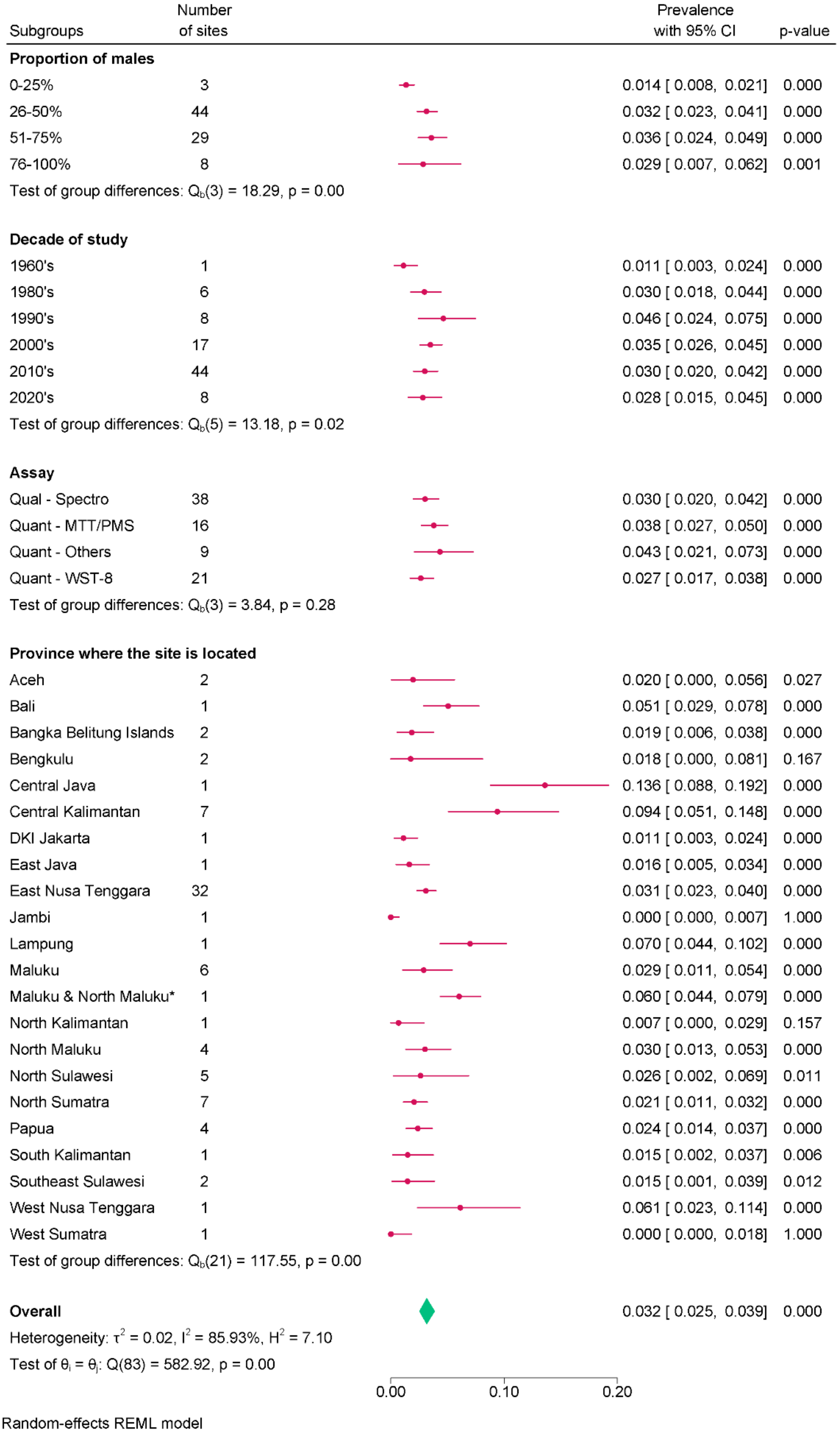

Supplementary Figure 3. Forest plot of the G6PD deficiency allele frequencies from eligible studies in Indonesia. Site identified the site name, and the Appendix reference number or local PI of the study where the data came from.

Site	n male deficient	n male total	Allele frequency with 95% CI	Weight (%)
Great Aceh[5]	2	32	0.063 [0.001, 0.179]	0.85
Middle Aceh[5]	1	56	0.018 [0.014, 0.075]	1.08
Afia[3]	2	53	0.038 [0.001, 0.110]	1.06
Boto Hilitano[3]	3	58	0.052 [0.007, 0.127]	1.09
Hiliana'a[3]	4	40	0.100 [0.023, 0.216]	0.94
Medan City[3,16]	9	133	0.068 [0.030, 0.117]	1.37
Durian[3]	1	37	0.027 [0.021, 0.112]	0.91
Perupuk & Guntung[3]	11	270	0.041 [0.020, 0.068]	1.51
Tanjung Leidong (Sutanto, 2016)	1	68	0.015 [0.011, 0.062]	1.15
Mentawai (Sudoyo, 2016)	0	94	0.000 [0.000, 0.018]	1.26
Bukit Dua Belas National Park (Sudoyo, 2015)	0	111	0.000 [0.000, 0.015]	1.32
Arga Makmur (Sudoyo, 2017)	0	86	0.000 [0.000, 0.020]	1.24
Enggano (Sudoyo, 2017)	0	63	0.000 [0.000, 0.027]	1.13
Bangka[15] (Satyagraha, 2013)	10	165	0.061 [0.029, 0.103]	1.42
Bangka Tengah(Satyagraha, 2013)	3	104	0.029 [0.004, 0.072]	1.30
Hanura (Sutanto, 2016)	16	115	0.139 [0.081, 0.209]	1.33
Jakarta[1]	5	446	0.011 [0.003, 0.024]	1.58
Semarang[14]	23	169	0.136 [0.088, 0.192]	1.42
Surabaya[10]	4	140	0.029 [0.006, 0.064]	1.38
Tenganan Pageringsingan[2]	16	166	0.096 [0.056, 0.146]	1.42
Waringin Agung (Syafruddin, 2014)	31	148	0.209 [0.147, 0.279]	1.39
Gunung Mas (Syafruddin, 2014)	8	145	0.055 [0.023, 0.099]	1.39
Palangkaraya[15]	3	50	0.060 [0.008, 0.147]	1.04
Sei Pinang (Syafruddin, 2014)	38	147	0.259 [0.191, 0.333]	1.39
Murung Raya (Syafruddin, 2014)	9	69	0.130 [0.060, 0.221]	1.16
Pujon (Syafruddin, 2014)	6	117	0.051 [0.017, 0.100]	1.33
Barito Utara (Syafruddin, 2014)	11	219	0.050 [0.025, 0.084]	1.48
2 cities in South Kalimantan (Satyagraha, 2012)	1	77	0.013 [0.010, 0.055]	1.20
Malinau[27,28]	0	45	0.000 [0.000, 0.038]	0.99
Lambuya Village[21]	1	45	0.022 [0.017, 0.093]	0.99
2 villages in Muna Island[21]	2	71	0.028 [0.000, 0.083]	1.17
2 Elementary Schools in Bolaang-Mongondow Regency[13]	6	89	0.067 [0.023, 0.130]	1.25
5 villages in Minahasa Regency[21]	2	158	0.013 [0.000, 0.038]	1.41
2 Elementary Schools in Minahasa Utara Regency[13]	10	104	0.096 [0.046, 0.161]	1.30
1 Elementary school in Bitung City[13]	0	50	0.000 [0.000, 0.034]	1.04
3 villages in Bangka Island[21]	4	22	0.182 [0.044, 0.375]	0.70
Sumbawa[6]	2	40	0.050 [0.001, 0.145]	0.94
Kodi[24]	24	143	0.168 [0.111, 0.234]	1.38
Panenggo Ede[25]	24	259	0.093 [0.060, 0.131]	1.51
Lamboya[24]	15	123	0.122 [0.069, 0.186]	1.34
Mata Pyawu[24]	1	59	0.017 [0.013, 0.071]	1.10
Mali Mada[24]	5	75	0.067 [0.019, 0.136]	1.19
Wanokaka[24]	6	155	0.039 [0.013, 0.076]	1.40
Anakalang[24]	2	61	0.033 [0.001, 0.096]	1.11
Wairasa[24]	1	66	0.015 [0.012, 0.064]	1.14
Umbu Ratu Nggay[24]	6	151	0.040 [0.013, 0.078]	1.40
4 villages near Labuan Bajo & Lembor[21]	1	159	0.006 [0.005, 0.027]	1.41
3 villages near Waingapu[6,22]	31	628	0.049 [0.034, 0.068]	1.61
3 districts in East Sumba[9,11]	16	193	0.083 [0.048, 0.126]	1.45
Tiworiwu Village[21]	3	72	0.042 [0.005, 0.103]	1.17
Reo Village[21]	6	54	0.111 [0.039, 0.211]	1.07
Tonggo Village[21]	1	49	0.020 [0.016, 0.086]	1.03
Ende[19]	7	177	0.040 [0.015, 0.074]	1.43
Reruwairere Village[21]	1	83	0.012 [0.009, 0.051]	1.22
Maumere[19]	31	465	0.067 [0.046, 0.091]	1.58
Sikka[19]	21	508	0.041 [0.026, 0.061]	1.59
Maumere & Talibura[18]	11	177	0.062 [0.031, 0.103]	1.43
Pruda Village[21]	18	132	0.136 [0.083, 0.201]	1.36
3 villages in Larantuka[21]	2	146	0.014 [0.000, 0.041]	1.39
Batu Putih[23]	2	61	0.033 [0.001, 0.096]	1.11
3 villages near Soe & Oebobo[21]	3	94	0.032 [0.004, 0.079]	1.26
Oenino[23]	1	44	0.023 [0.018, 0.095]	0.98
Alor[6]	4	60	0.067 [0.015, 0.146]	1.11
Oe'ekam[23]	0	42	0.000 [0.000, 0.041]	0.96
Panite[23]	4	45	0.089 [0.020, 0.193]	0.99
Oinlasi[23]	0	35	0.000 [0.000, 0.049]	0.89
Boking (Noviyanti, 2018)	2	165	0.012 [0.000, 0.036]	1.42
Siokona[7]	1	41	0.024 [0.019, 0.102]	0.95
Oba[7]	22	428	0.051 [0.032, 0.075]	1.58
Oba Selatan[7]	8	85	0.094 [0.040, 0.167]	1.23
Maba (Satyagraha, 2012)	2	65	0.031 [0.001, 0.091]	1.14
Buru & Halmahera[17]	42	696	0.060 [0.044, 0.079]	1.62
Pulau Romang[20]	2	40	0.050 [0.001, 0.145]	0.94
Pulau Babar[20]	3	27	0.111 [0.015, 0.263]	0.78
Seram Utara (Sudoyo, 2017)	0	55	0.000 [0.000, 0.031]	1.07
Saumlaki[20]	1	26	0.038 [0.030, 0.158]	0.77
Larat[20]	1	31	0.032 [0.025, 0.133]	0.84
Pulau Kur[20]	1	20	0.050 [0.040, 0.202]	0.66
Timika (Poespoprodjo, 2018 & Satyagraha, 2020)	11	317	0.035 [0.017, 0.058]	1.54
Arso PIR[4]	4	179	0.022 [0.005, 0.050]	1.44
Arso XI[5]	2	131	0.015 [0.000, 0.045]	1.36
Waris (Syafruddin, 2017)	5	56	0.089 [0.026, 0.180]	1.08
**Overall**			0.045 [0.035, 0.055]	

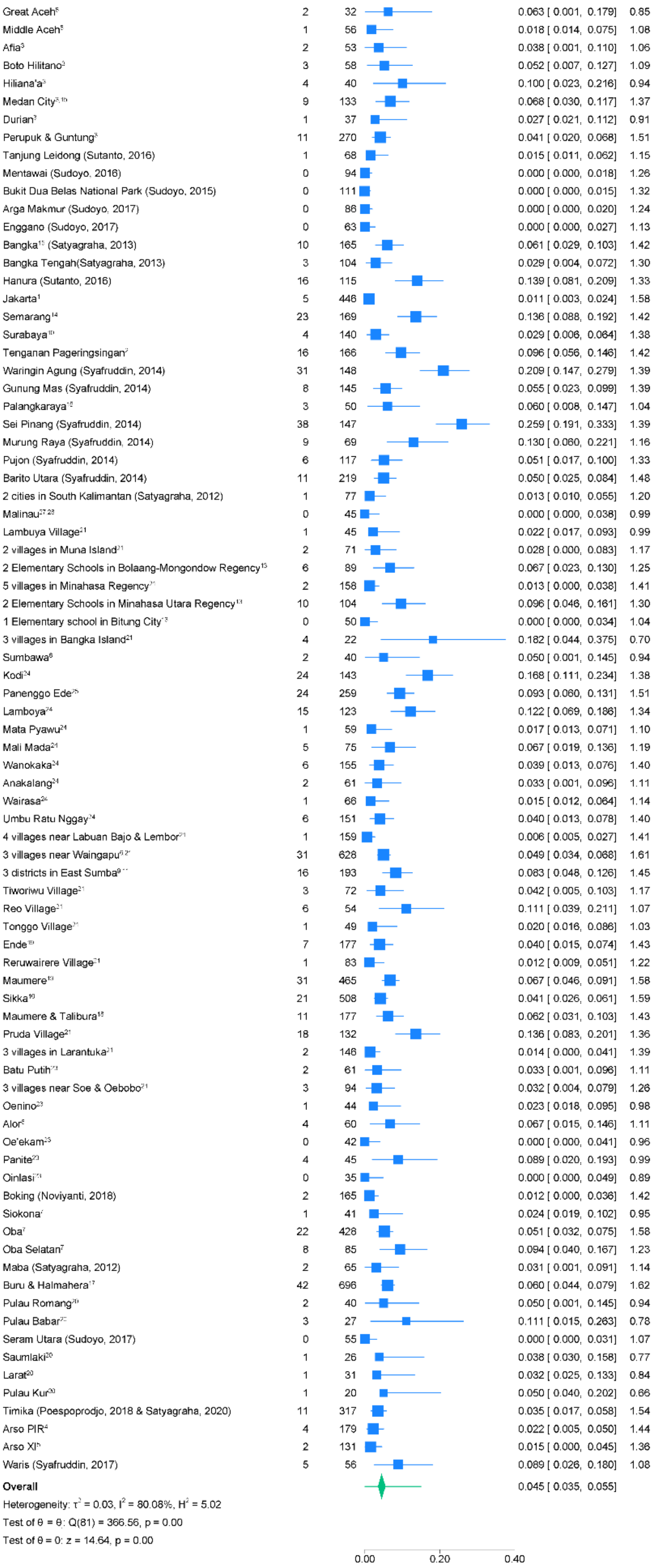

Heterogeneity: $\tau^2 = 0.03$, $I^2 = 80.08\%$, $H^2 = 5.02$

Test of $\theta_i = \theta_j$: $Q(81) = 366.56$, $p = 0.00$

Test of $\theta = 0$: $z = 14.64$, $p = 0.00$

Random-effects REML model
95% prediction interval

Supplementary Figure 4. Forest plot of the prevalence of female participants with G6PD activity <70% of normal from eligible studies in Indonesia. Site identified the site name, and the Appendix reference number or local PI of the study where the data came from. *(Sudoyo, 2015); **(Satyagraha, 2012); ***(Poespoprodjo, 2018 & Satyagraha, 2020).

Study	n female with <70% activity	n female total	Prevalence with 95% CI	Weight (%)
Tanjung Leidong (Sutanto, 2016)	7	240	0.029 [0.011, 0.055]	3.00
Bukit Dua Belas National Park*	1	128	0.008 [0.006, 0.033]	2.87
Arga Makmur (Sudoyo, 2017)	10	242	0.041 [0.019, 0.071]	3.00
Enggano (Sudoyo, 2017)	7	92	0.076 [0.029, 0.140]	2.77
Bangka (Satyagraha, 2013)	11	203	0.054 [0.027, 0.090]	2.97
Bangka Tengah (Satyagraha, 2013)	4	178	0.022 [0.005, 0.051]	2.95
Hanura (Sutanto, 2016)	27	185	0.146 [0.098, 0.201]	2.95
Waringin Agung (Syafruddin, 2014)	36	145	0.248 [0.181, 0.322]	2.90
Gunung Mas (Syafruddin, 2014)	28	85	0.329 [0.233, 0.433]	2.75
Sei Pinang (Syafruddin, 2014)	72	200	0.360 [0.295, 0.428]	2.97
Murung Raya (Syafruddin, 2014)	9	77	0.117 [0.053, 0.199]	2.71
Pujon (Syafruddin, 2014)	10	66	0.152 [0.074, 0.249]	2.65
Barito Utara (Syafruddin, 2014)	50	112	0.446 [0.355, 0.540]	2.84
2 cities in South Kalimantan**	26	124	0.210 [0.142, 0.286]	2.86
Malinau[27,28]	4	100	0.040 [0.009, 0.089]	2.80
Kodi[24]	31	188	0.165 [0.115, 0.222]	2.96
Kodi Balaghar[26]	160	1,350	0.119 [0.102, 0.136]	3.13
Umbu Ngedo[26]	89	678	0.131 [0.107, 0.158]	3.10
Panenggo Ede[25]	38	348	0.109 [0.078, 0.144]	3.05
Lamboya[24]	27	193	0.140 [0.094, 0.193]	2.96
Mata Pyawu[24]	2	93	0.022 [0.000, 0.064]	2.78
Mali Mada[24]	7	96	0.073 [0.028, 0.135]	2.79
Wanokaka[24]	9	203	0.044 [0.020, 0.078]	2.97
Anakalang[24]	6	102	0.059 [0.020, 0.114]	2.81
Wairasa[24]	6	119	0.050 [0.017, 0.098]	2.85
Umbu Ratu Nggay[24]	8	169	0.047 [0.020, 0.085]	2.94
Batu Putih[23]	11	58	0.190 [0.098, 0.302]	2.59
Oenino[23]	7	90	0.078 [0.030, 0.144]	2.77
Oe'ekam[23]	4	58	0.069 [0.015, 0.151]	2.59
Panite[23]	6	54	0.111 [0.039, 0.211]	2.56
Oinlasi[23]	18	65	0.277 [0.174, 0.393]	2.64
Boking (Noviyanti, 2018)	7	129	0.054 [0.021, 0.101]	2.87
Maba (Satyagraha, 2012)	2	75	0.027 [0.000, 0.079]	2.70
Timika***	25	334	0.075 [0.049, 0.106]	3.04
Waris (Syafruddin, 2017)	13	150	0.087 [0.046, 0.138]	2.91
**Overall**			0.104 [0.076, 0.137]	

Heterogeneity: $\tau^2 = 0.08$, $I^2 = 93.52\%$, $H^2 = 15.43$

Test of $\theta_i = \theta_j$: $Q(34) = 397.73$, $p = 0.00$

Test of $\theta = 0$: $z = 11.60$, $p = 0.00$

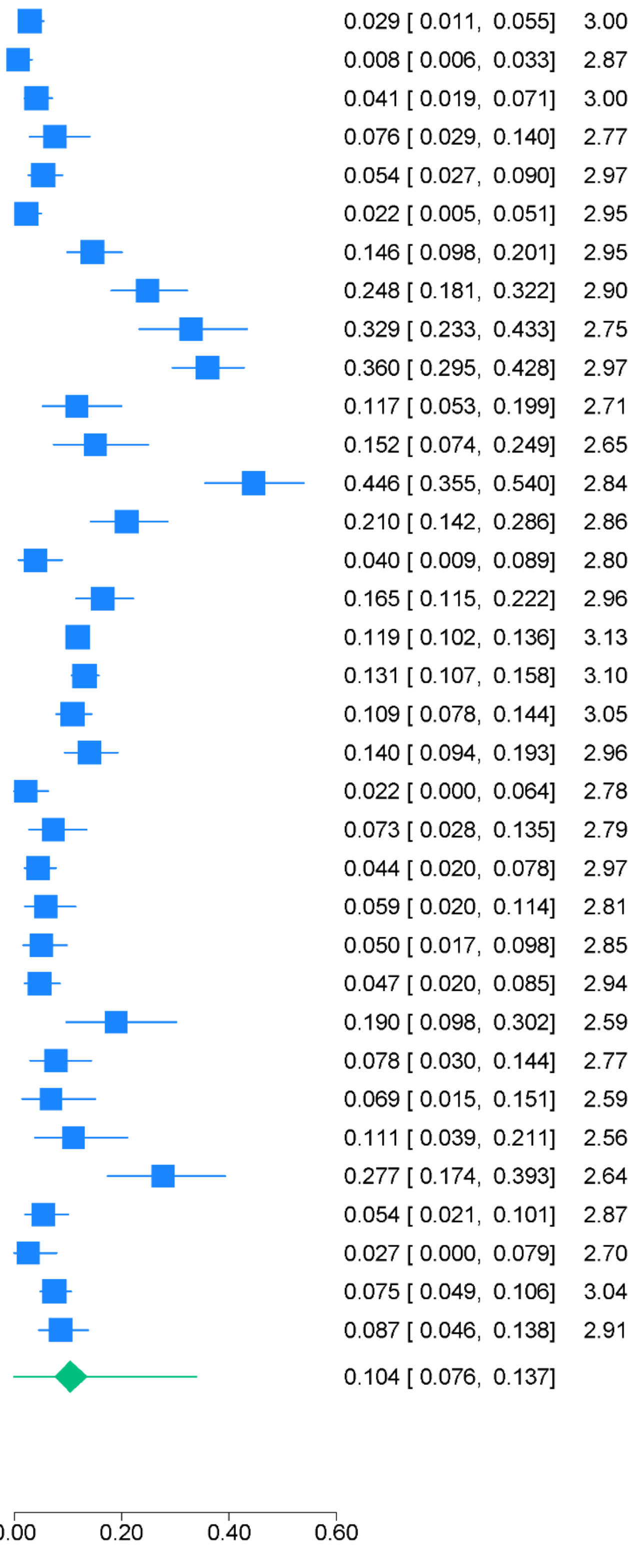

Random-effects REML model
95% prediction interval

Supplementary Figure 5. A funnel plot visualising the relationship between site-specific effect sizes (Freeman-Tukey's p) and their precision (Standard error) of all included studies. The data is presented by site, there was 87 sites in total.

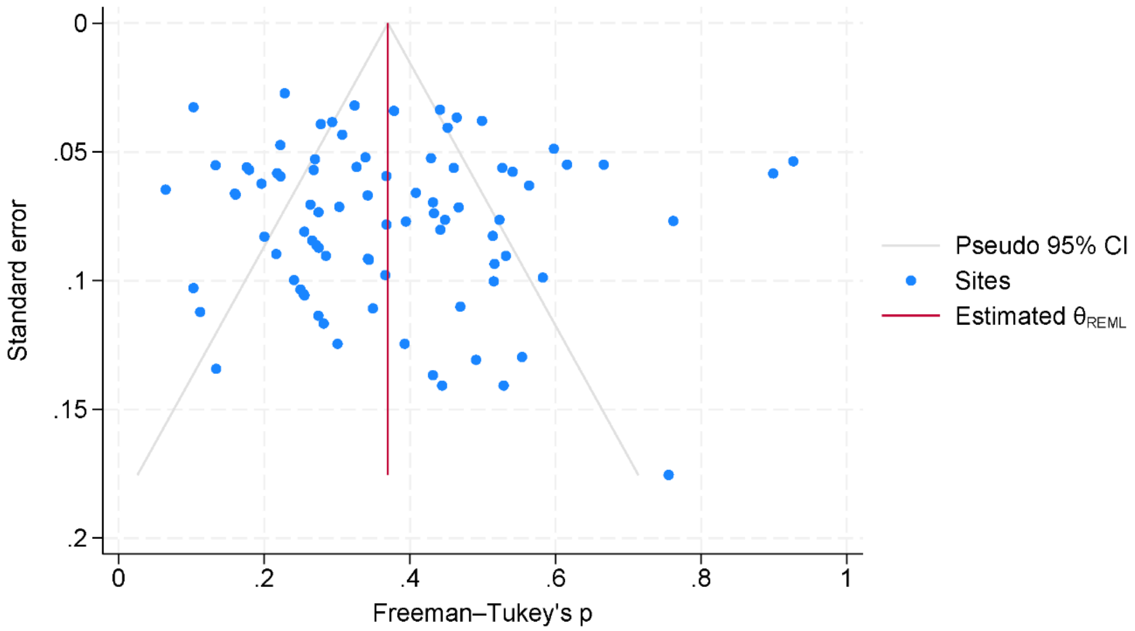

Supplementary Figure 6. Summary table of the leave-one-out sensitivity analysis of the prevalence of G6PD deficiency from eligible studies in Indonesia (87 sites). The displayed Proportion corresponds to the pooled prevalence computed from a meta-analysis excluding that site; if its 95% confidence interval contains the pooled prevalence based on all sites (0.031), that site does not exert a significantly larger influence on the pooled prevalence.

```
  Effect-size label: Freeman–Tukey's p
        Effect size: _meta_es
          Std. err.: _meta_se
        Study label: site_name

Leave-one-out meta-analysis summary                  Number of studies =     87
Random-effects model
Method: REML
```

Omitted study	Proportion	[95% conf.	interval]	p-value
14 villages in Sabang City	0.031	0.025	0.038	0.000
Great Aceh	0.031	0.024	0.038	0.000
Middle Aceh	0.031	0.025	0.038	0.000
Afia	0.031	0.024	0.038	0.000
Boto Hilitano	0.031	0.024	0.038	0.000
Hiliana'a	0.030	0.024	0.037	0.000
Medan City	0.031	0.024	0.038	0.000
Durian	0.031	0.024	0.038	0.000
Perupuk & Guntung	0.031	0.024	0.038	0.000
Tanjung Leidong	0.031	0.025	0.038	0.000
Mentawai	0.031	0.025	0.038	0.000
Bukit Dua Belas National Park	0.031	0.025	0.038	0.000
Arga Makmur	0.031	0.025	0.038	0.000
Enggano	0.030	0.024	0.037	0.000
Bangka	0.031	0.024	0.038	0.000
Bangka Tengah	0.031	0.025	0.038	0.000
Hanura	0.030	0.024	0.037	0.000
Jakarta	0.031	0.025	0.038	0.000
Menoreh Hills	0.031	0.025	0.038	0.000
Semarang	0.030	0.024	0.037	0.000
Surabaya	0.031	0.024	0.038	0.000
Tenganan Pageringsingan	0.030	0.024	0.037	0.000
Waringin Agung	0.029	0.024	0.036	0.000
Gunung Mas	0.031	0.024	0.038	0.000
Palangkaraya	0.030	0.024	0.037	0.000
Sei Pinang	0.029	0.023	0.036	0.000
Murung Raya	0.030	0.024	0.037	0.000
Pujon	0.030	0.024	0.037	0.000
Barito Utara	0.030	0.024	0.037	0.000
2 cities in South Kalimantan	0.031	0.024	0.038	0.000
Malinau	0.031	0.025	0.038	0.000
Lambuya Village	0.031	0.024	0.038	0.000
2 villages in Muna Island	0.031	0.024	0.038	0.000
2 Elementary Schools in Bolaang-Mongondow Regency	0.031	0.024	0.038	0.000
5 villages in Minahasa Regency	0.031	0.025	0.038	0.000
2 Elementary Schools in Minahasa Utara Regency	0.030	0.024	0.037	0.000
1 Elementary school in Bitung City	0.031	0.025	0.038	0.000
3 villages in Bangka Island	0.030	0.024	0.037	0.000
Sumbawa	0.030	0.024	0.037	0.000
Kodi	0.030	0.024	0.037	0.000
Kodi Balaghar	0.031	0.025	0.038	0.000
Umbu Ngedo	0.031	0.024	0.038	0.000
Panenggo Ede	0.030	0.024	0.037	0.000
Lamboya	0.030	0.024	0.037	0.000
Mata Pyawu	0.031	0.025	0.038	0.000
Mali Mada	0.030	0.024	0.037	0.000
Wanokaka	0.031	0.024	0.038	0.000
Anakalang	0.031	0.024	0.038	0.000
Wairasa	0.031	0.024	0.038	0.000
Umbu Ratu Nggay	0.031	0.024	0.038	0.000
4 villages near Labuan Bajo & Lembor	0.031	0.025	0.038	0.000
3 villages near Waingapu	0.030	0.024	0.037	0.000
3 districts in East Sumba	0.030	0.024	0.037	0.000
Tiworiwu Village	0.031	0.024	0.038	0.000
Reo Village	0.030	0.024	0.037	0.000
Tonggo Village	0.031	0.025	0.038	0.000
Ende	0.030	0.024	0.037	0.000
Reruwairere Village	0.031	0.025	0.038	0.000
Maumere	0.030	0.024	0.037	0.000
Sikka	0.031	0.024	0.038	0.000
Maumere & Talibura	0.030	0.024	0.037	0.000
Pruda Village	0.030	0.024	0.037	0.000
3 villages in Larantuka	0.031	0.025	0.038	0.000
Batu Putih	0.031	0.024	0.038	0.000
3 villages near Soe & Oebobo	0.031	0.024	0.038	0.000
Oenino	0.031	0.024	0.038	0.000
Alor	0.030	0.024	0.037	0.000
Oe'ekam	0.031	0.025	0.038	0.000
Panite	0.030	0.024	0.037	0.000
Oinlasi	0.031	0.025	0.038	0.000
Insana	0.031	0.024	0.038	0.000
Boking	0.031	0.025	0.038	0.000
Siokona	0.031	0.025	0.038	0.000
Oba	0.031	0.024	0.038	0.000
Oba Selatan	0.030	0.024	0.037	0.000
Maba	0.031	0.024	0.038	0.000
Buru & Halmahera	0.030	0.024	0.037	0.000
Pulau Romang	0.031	0.024	0.038	0.000
Pulau Babar	0.031	0.024	0.037	0.000
Seram Utara	0.031	0.025	0.038	0.000
Saumlaki	0.031	0.024	0.038	0.000
Larat	0.030	0.024	0.037	0.000
Pulau Kur	0.031	0.024	0.038	0.000
Timika	0.031	0.024	0.038	0.000
Arso PIR	0.031	0.024	0.038	0.000
Arso XI	0.031	0.024	0.038	0.000
Waris	0.030	0.024	0.037	0.000
Proportion	0.031	0.024	0.038	0.000

Supplementary Figure 7. Summary table of the leave-one-out sensitivity analysis of the G6PD deficiency allele frequencies from eligible studies in Indonesia (82 sites). The displayed Proportion corresponds to the pooled allele frequency computed from a meta-analysis excluding that site; if its 95% confidence interval contains the pooled allele frequency based on all sites (0.045), that site does not exert a significantly larger influence on the pooled allele frequency.

```
  Effect-size label: Freeman–Tukey's p
        Effect size: _meta_es
          Std. err.: _meta_se
        Study label: site_name

Leave-one-out meta-analysis summary                 Number of studies =     82
Random-effects model
Method: REML
```

Omitted study	Proportion	[95% conf.	interval]	p-value
Great Aceh	0.044	0.035	0.055	0.000
Middle Aceh	0.045	0.035	0.055	0.000
Afia	0.045	0.035	0.055	0.000
Boto Hilitano	0.045	0.035	0.055	0.000
Hiliana'a	0.044	0.035	0.055	0.000
Medan City	0.044	0.035	0.055	0.000
Durian	0.045	0.035	0.055	0.000
Perupuk & Guntung	0.045	0.035	0.055	0.000
Tanjung Leidong	0.045	0.035	0.056	0.000
Mentawai	0.046	0.036	0.056	0.000
Bukit Dua Belas National Park	0.046	0.036	0.056	0.000
Arga Makmur	0.046	0.036	0.056	0.000
Enggano	0.045	0.036	0.056	0.000
Bangka	0.044	0.035	0.055	0.000
Bangka Tengah	0.045	0.035	0.055	0.000
Hanura	0.044	0.034	0.054	0.000
Jakarta	0.045	0.036	0.056	0.000
Semarang	0.044	0.034	0.054	0.000
Surabaya	0.045	0.035	0.055	0.000
Tenganan Pageringsingan	0.044	0.034	0.054	0.000
Waringin Agung	0.043	0.034	0.053	0.000
Gunung Mas	0.044	0.035	0.055	0.000
Palangkaraya	0.044	0.035	0.055	0.000
Sei Pinang	0.043	0.034	0.052	0.000
Murung Raya	0.044	0.034	0.054	0.000
Pujon	0.044	0.035	0.055	0.000
Barito Utara	0.045	0.035	0.055	0.000
2 cities in South Kalimantan	0.045	0.035	0.056	0.000
Malinau	0.045	0.036	0.056	0.000
Lambuya Village	0.045	0.035	0.055	0.000
2 villages in Muna Island	0.045	0.035	0.055	0.000
2 Elementary Schools in Bolaang-Mongondow Regency	0.044	0.035	0.055	0.000
5 villages in Minahasa Regency	0.045	0.036	0.056	0.000
2 Elementary Schools in Minahasa Utara Regency	0.044	0.035	0.054	0.000
1 Elementary school in Bitung City	0.045	0.036	0.056	0.000
3 villages in Bangka Island	0.044	0.035	0.054	0.000
Sumbawa	0.045	0.035	0.055	0.000
Kodi	0.043	0.034	0.054	0.000
Panenggo Ede	0.044	0.034	0.054	0.000
Lamboya	0.044	0.034	0.054	0.000
Mata Pyawu	0.045	0.035	0.056	0.000
Mali Mada	0.044	0.035	0.055	0.000
Wanokaka	0.045	0.035	0.055	0.000
Anakalang	0.045	0.035	0.055	0.000
Wairasa	0.045	0.035	0.056	0.000
Umbu Ratu Nggay	0.045	0.035	0.055	0.000
4 villages near Labuan Bajo & Lembor	0.045	0.036	0.056	0.000
3 villages near Waingapu	0.045	0.035	0.055	0.000
3 districts in East Sumba	0.044	0.035	0.055	0.000
Tiworiwu Village	0.045	0.035	0.055	0.000
Reo Village	0.044	0.035	0.054	0.000
Tonggo Village	0.045	0.035	0.055	0.000
Ende	0.045	0.035	0.055	0.000
Reruwairere Village	0.045	0.035	0.056	0.000
Maumere	0.044	0.035	0.055	0.000
Sikka	0.045	0.035	0.055	0.000
Maumere & Talibura	0.044	0.035	0.055	0.000
Pruda Village	0.044	0.034	0.054	0.000
3 villages in Larantuka	0.045	0.036	0.056	0.000
Batu Putih	0.045	0.035	0.055	0.000
3 villages near Soe & Oebobo	0.045	0.035	0.055	0.000
Oenino	0.045	0.035	0.055	0.000
Alor	0.044	0.035	0.055	0.000
Oe'ekam	0.045	0.036	0.056	0.000
Panite	0.044	0.035	0.055	0.000
Oinlasi	0.045	0.036	0.056	0.000
Boking	0.045	0.036	0.056	0.000
Siokona	0.045	0.035	0.055	0.000
Oba	0.044	0.035	0.055	0.000
Oba Selatan	0.044	0.035	0.055	0.000
Maba	0.045	0.035	0.055	0.000
Buru & Halmahera	0.044	0.035	0.055	0.000
Pulau Romang	0.045	0.035	0.055	0.000
Pulau Babar	0.044	0.035	0.055	0.000
Seram Utara	0.045	0.036	0.056	0.000
Saumlaki	0.045	0.035	0.055	0.000
Larat	0.045	0.035	0.055	0.000
Pulau Kur	0.045	0.035	0.055	0.000
Timika	0.045	0.035	0.055	0.000
Arso PIR	0.045	0.035	0.056	0.000
Arso XI	0.045	0.035	0.056	0.000
Waris	0.044	0.035	0.055	0.000
Proportion	0.045	0.035	0.055	0.000

Supplementary Figure 8. Summary table of the leave-one-out sensitivity analysis of the prevalence of female participants with G6PD activity <70% of normal from eligible studies in Indonesia (35 sites). The displayed Proportion corresponds to the pooled prevalence computed from a meta-analysis excluding that site; if its 95% confidence interval contains the pooled prevalence based on all sites (0.104), that site does not exert a significantly larger influence on the pooled prevalence.

```
  Effect-size label: Freeman-Tukey's p
        Effect size: _meta_es
          Std. err.: _meta_se
        Study label: site_name

Leave-one-out meta-analysis summary              Number of studies =     35
Random-effects model
Method: REML
```

Omitted study	Proportion	[95% conf.	interval]	p-value
Tanjung Leidong	0.107	0.078	0.140	0.000
Bukit Dua Belas National Park	0.108	0.080	0.141	0.000
Arga Makmur	0.107	0.077	0.140	0.000
Enggano	0.105	0.076	0.138	0.000
Bangka	0.106	0.077	0.139	0.000
Bangka Tengah	0.108	0.078	0.140	0.000
Hanura	0.103	0.074	0.136	0.000
Waringin Agung	0.101	0.073	0.133	0.000
Gunung Mas	0.099	0.072	0.130	0.000
Sei Pinang	0.098	0.072	0.128	0.000
Murung Raya	0.104	0.075	0.137	0.000
Pujon	0.103	0.074	0.136	0.000
Barito Utara	0.097	0.072	0.125	0.000
2 cities in South Kalimantan	0.102	0.073	0.134	0.000
Malinau	0.107	0.077	0.140	0.000
Kodi	0.103	0.074	0.136	0.000
Kodi Balaghar	0.104	0.075	0.137	0.000
Umbu Ngedo	0.103	0.074	0.137	0.000
Panenggo Ede	0.104	0.075	0.137	0.000
Lamboya	0.103	0.074	0.136	0.000
Mata Pyawu	0.107	0.078	0.140	0.000
Mali Mada	0.105	0.076	0.139	0.000
Wanokaka	0.106	0.077	0.140	0.000
Anakalang	0.106	0.076	0.139	0.000
Wairasa	0.106	0.077	0.139	0.000
Umbu Ratu Nggay	0.106	0.077	0.140	0.000
Batu Putih	0.102	0.074	0.135	0.000
Oenino	0.105	0.076	0.138	0.000
Oe'ekam	0.105	0.076	0.139	0.000
Panite	0.104	0.075	0.137	0.000
Oinlasi	0.101	0.073	0.132	0.000
Boking	0.106	0.077	0.139	0.000
Maba	0.107	0.078	0.140	0.000
Timika	0.105	0.076	0.139	0.000
Waris	0.105	0.075	0.138	0.000
Proportion	0.104	0.076	0.137	0.000

Supplementary Figure 9. Geostatistical map of lower (A) and upper (B) limits of predicted G6PD deficiency prevalence in Indonesia modelled from site-specific G6PD deficiency prevalence data. Black dots mark study sites.

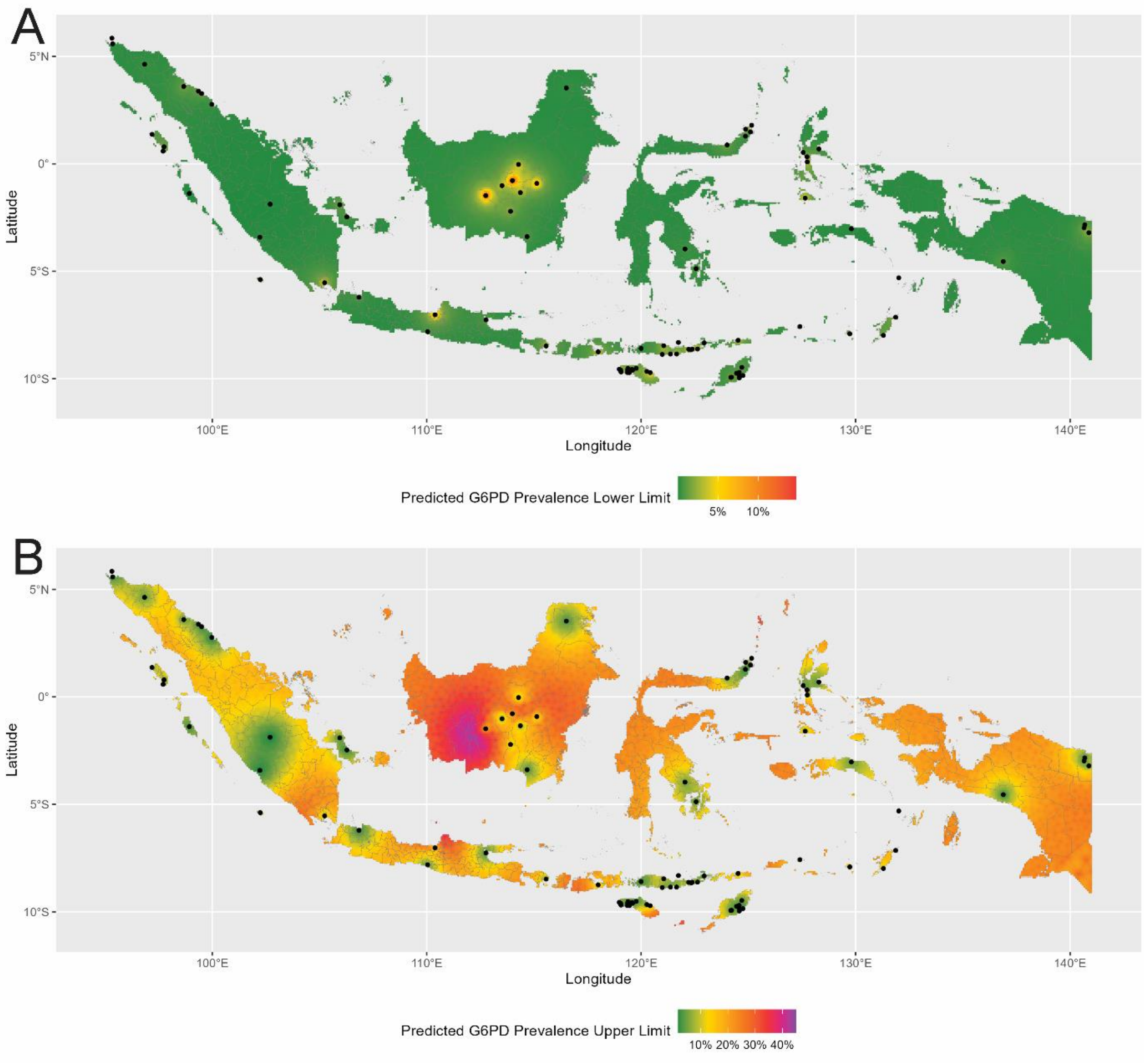

Supplementary Figure 10. Histograms showing the distribution of Conditional Predictive Ordinate (CPO) values (A) and Probability Integral Transform (PIT) values (B) for each observation in the geostatistical model of G6PDd prevalence. Small CPO values indicate that the model fits the observations poorly, and the non-uniform distribution of PIT values further indicates that the model does not represent the observations well.

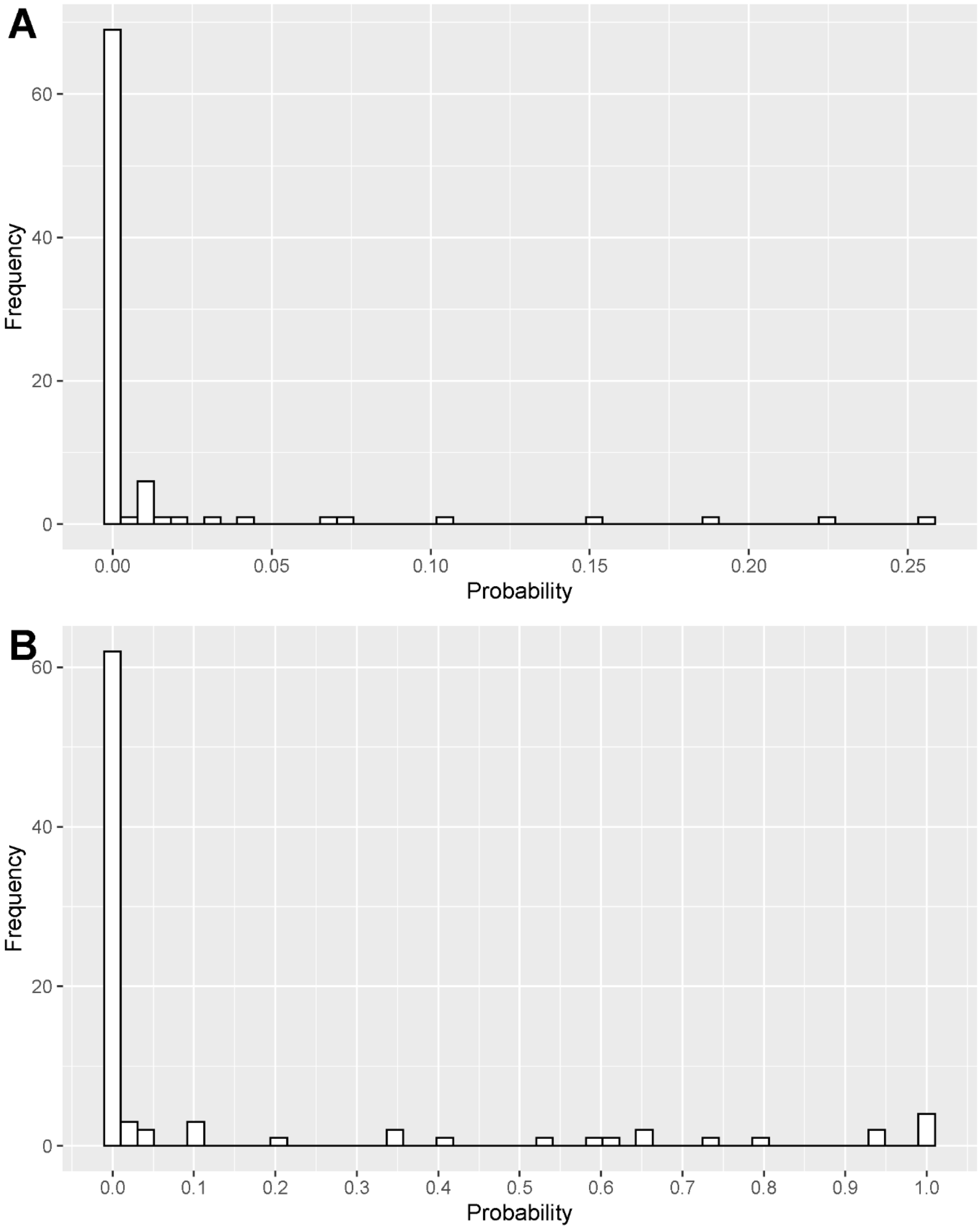

Supplementary Figure 11. Scatterplot showing the correlation between G6PDd prevalence and allele frequency among study sites with male participants.

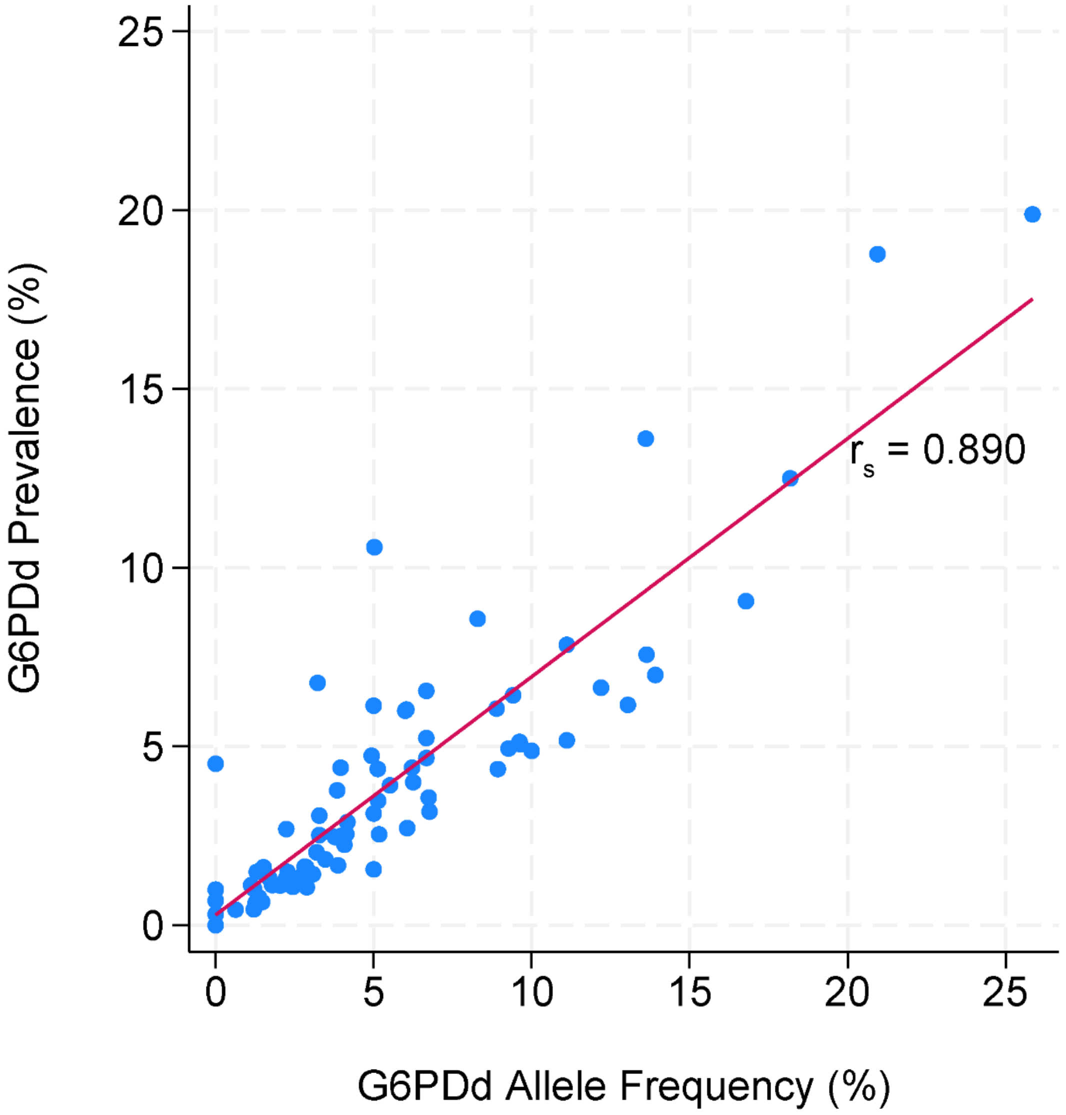

References

1. Eng L-IL, Giok P-OH. Glucose-6-phosphate Dehydrogenase Deficiency in Indonesia. *Nature* 1964; **204**(4953): 88–9.
2. Breguet G, Ney R, Kirk RL, Blake NM. Genetic Survey of an Isolated Community in Bali, Indonesia. 1982: 10.
3. Matsuoka H, Ishii A, Panjaitan W, Sudiranto R. Malaria and glucose-6-phosphate dehydrogenase deficiency in North Sumatra, Indonesia. *The Southeast Asian Journal of Tropical Medicine and Public Health* 1986; **17**(4): 530–6.
4. Jones T, Baird J, Ratiwayanto S, Supriatman M. Glucose-6-Phosphate Dehydrogenase Deficiency and Haemoglobinophaties in Resident of Arso PIR, Irian Jaya. *Bulletin of Health Research* 1990; **18**: 9.
5. Fryauff D, Baird K, Basri H, et al. Randomised placebo-controlled trial of primaquine for prophylaxis of falciparum and vivax malaria. *The Lancet* 1995; **346**(8984): 1190–3.
6. Azhar A. Kajian Genetika Biokemis Dehidrogenase Glukosa-6-Fosfat (G6PD) dan Dehidrogenase 6-Fosfoglukonat (6-PGD) pada Tiga Populasi Nusa Tenggara. Yogyakarta: Universitas Gadjah Mada; 1998.
7. Tantular IS, Iwai K, Lin K, et al. Field trials of a rapid test for G6PD deficiency in combination with a rapid diagnosis of malaria. *Tropical Medicine & International Health* 1999; **4**(4): 245–50.
8. Azhar A, Husin A. Prevalence of glucose 6-phosphate dehydro(G6PD) deficiency in two populations of Aceh province. *Jurnal Kedokteran YARSI* 2001; **9**(1): 93–5.
9. Syahyuni R. Hubungan Defisiensi Glucose-6-Phosphate Dehydrogenase (G-6-PD) dengan Kepadatan Parasit Malaria pada Anak Usia Sekolah di Daerah Endemis Malaria. Semarang: Universitas Diponegoro; 2003.
10. Jalloh A, Tantular IS, Pusarawati S, et al. Rapid epidemiologic assessment of glucose-6-phosphate dehydrogenase deficiency in malaria-endemic areas in Southeast Asia using a novel diagnostic kit. *Tropical Medicine & International Health* 2004; **9**(5): 615–23.
11. Shimizu H, Tamam M, Soemantri A, Ishida T. Glucose-6-phosphate dehydrogenase deficiency and Southeast Asian ovalocytosis in asymptomatic Plasmodium carriers in Sumba island, Indonesia. *Journal of Human Genetics* 2005; **50**(8): 420–4.
12. Lederman ER, Maguire JD, Sumawinata IW, et al. Combined chloroquine, sulfadoxine/pyrimethamine and primaquine against Plasmodium falciparum in Central Java, Indonesia. *Malar J* 2006; **5**: 108.
13. Tuda JSB, Kepel BJ, Nakatsu M, Matsuoka H. Prevalensi defisiensi Glucose-6-Phosphate Dehydrogenase (G6PD) pada anak Sekolah Dasar yang tinggal di daerah endemis malaria di Sulawesi utara. *YARSI Medical Journal* 2007; **15**(1).
14. Soemantri A, Saha S, Saha N, Tay JSH. Molecular Variants of Red Cell Glucose-6-Phosphate Dehydrogenase Deficiency in Central Java, Indonesia. *Human Heredity* 1995; **45**(6).
15. Davy MRFM. Molecular Analysis for the Detection of Glucose-6-Phosphate Dehydrogenase (G6PD) Deficiency. Yogyakarta: Universitas Gadjah Mada; 2000.
16. Hardjowasito W, Pardjianto B, Fitri LE, et al. Identification of point mutations in Glucose-6-Phosphate Dehydrogenase gene in Timor Island people : A preliminary report. *Medical Journal of Indonesia; Jakarta* 2001; **10**(4): 210–3.
17. Iwai K, Hirono A, Matsuoka H, et al. Distribution of glucose-6-phosphate dehydrogenase mutations in Southeast Asia. *Human Genetics* 2001; **108**(6): 445–9.
18. Matsuoka H, Arai M, Yoshida S, et al. Five different glucose-6-phophate dehydrogenase (G6PD) variants found among 11 G6PD-deficient persons in Flores Island, Indonesia. *Journal of Human Genetics* 2003; **48**(10): 541–4.
19. Kawamoto F, Matsuoka H, Kanbe T, et al. Further investigations of glucose-6-phosphate dehydrogenase variants in Flores Island, eastern Indonesia. *Journal of Human Genetics* 2006; **51**(11): 952–7.
20. Suhartati. Mutasi Gen Penyebab Defisiensi Glukosa 6 Fosfat Dehidrogenase (G6PD) di Surabaya dan Kepulauan Maluku Tenggara. Surabaya: Universitas Airlangga; 2006.
21. Tantular IS, Matsuoka H, Kasahara Y, et al. Incidence and Mutation Analysis of Glucose-6-Phosphate Dehydrogenase Deficiency in Eastern Indonesian Populations. *Acta Med Okayama* 2010; **64**(6): 7.
22. Asih PB, Rozi IE, Herdiana, et al. The baseline distribution of malaria in the initial phase of elimination in Sabang Municipality, Aceh Province, Indonesia. *Malaria Journal* 2012; **11**(1): 291.
23. Hutagalung J, Kusnanto H, Supargiyono S, et al. The first evaluation of glucose-6-phospate dehydrogenase defciency (G6PD) gene mutation in malaria endemic region at South Central Timor (SCT) district, Eastern Indonesia 2014–2015. *Indonesian Journal of Biotechnology* 2015; **20**(2): 117–32.
24. Satyagraha AW, Sadhewa A, Baramuli V, et al. G6PD Deficiency at Sumba in Eastern Indonesia Is Prevalent, Diverse and Severe: Implications for Primaquine Therapy against Relapsing Vivax Malaria. *PLOS Neglected Tropical Diseases* 2015; **9**(3): e0003602.

25. Satyagraha AW, Sadhewa A, Elvira R, et al. Assessment of Point-of-Care Diagnostics for G6PD Deficiency in Malaria Endemic Rural Eastern Indonesia. *PLOS Neglected Tropical Diseases* 2016; **10**(2): e0004457.
26. Satyagraha AW, Sadhewa A, Panggalo LV, et al. Genotypes and phenotypes of G6PD deficiency among Indonesian females across diagnostic thresholds of G6PD activity guiding safe primaquine therapy of latent malaria. *PLOS Neglected Tropical Diseases* 2021; **15**(7): e0009610.
27. Sadhewa A, Chaudhary A, Panggalo LV, et al. Field assessment of the operating procedures of a semi-quantitative G6PD Biosensor to improve repeatability of routine testing. *PLoS One* 2024; **19**(1): e0296708.
28. Sadhewa A, Panggalo LV, Nanine I, et al. Field evaluation of a novel semi-quantitative point-of-care diagnostic for G6PD deficiency in Indonesia. *PLoS One* 2024; **19**(4): e0301506.
